# Metabolomic Analysis Reveals Vitamin D-induced Decrease in Polyol Pathway and Subtle Modulation of Glycolysis in HEK293T Cells

**DOI:** 10.1038/s41598-017-10006-9

**Published:** 2017-08-25

**Authors:** G. C. Santos, J. D. Zeidler, J. A. Pérez-Valencia, A. C. B. Sant’Anna-Silva, A. T. Da Poian, T. El-Bacha, F. C. L. Almeida

**Affiliations:** 1 Federal University of Rio de Janeiro, Institute of Medical Biochemistry, Leopoldo de Meis, Brazil; 2 Federal University of Rio de Janeiro, National Center for Structural Biology and Bioimaging (CENABIO)/National Center for Nuclear Magnetic Resonance (CNRMN), Rio de Janeiro, Brazil; 3 Federal University of Rio de Janeiro, Institute of Nutrition Josué de Castro, Rio de Janeiro, Brazil

## Abstract

We combined ^1^H NMR metabolomics with functional and molecular biochemical assays to describe the metabolic changes elicited by vitamin D in HEK293T, an embryonic proliferative cell line adapted to high-glucose concentrations. Activation of the polyol pathway, was the most important consequence of cell exposure to high glucose concentration, resembling cells exposed to hyperglycemia. Vitamin D induced alterations in HEK293T cells metabolism, including a decrease in sorbitol, glycine, glutamate, guanine. Vitamin D modulated glycolysis by increasing phosphoglycerate mutase and decreasing enolase activities, changing carbon fate without changing glucose consumption, lactate export and Krebs cycle. The decrease in sorbitol intracellular concentration seems to be related to vitamin D regulated redox homeostasis and protection against oxidative stress, and helped maintaining the high proliferative phenotype, supported by the decrease in glycine and guanine and orotate concentration and increase in choline and phosphocholine concentration. The decrease in orotate and guanine indicated an increased biosynthesis of purine and pyrimidines. Vitamin D elicited metabolic alteration without changing cellular proliferation and mitochondrial respiration, but reclaiming reductive power. Our study may contribute to the understanding of the metabolic mechanism of vitamin D upon exposure to hyperglycemia, suggesting a role of protection against oxidative stress.

## Introduction

1,25-dihydroxycholecalciferol (1,25(OH)_2_D3; vitamin D) is a hormone that has a plethora of biological effects, such as regulation of calcium and phosphate homeostasis^1–3^, immune response^4, 5^, and anti-cancer related effects, including inhibition of cell proliferation^6, 7^, invasive potential^8^ and metastasis^9^. It has been shown that vitamin D treatment inhibits glycolytic flux in metastatic *ras* transformed MCF10A cells, as shown by a decrease in 3-phosphoglycerate and lactate contents and a reduced activity of lactate dehydrogenase^10^. In the same cells, vitamin D inhibits glutamine metabolism, reducing glutamate and glutamine intracellular contents^11^. Additionally, vitamin D treatment increased insulin secretion in polycystic ovary syndrome (PCOS) patients^12^, and suppressed the expression of angiotensinogen induced by hyperglycemia by blocking NF-kB-mediated pathway^13^. These results show that vitamin D regulates several metabolic pathways and cell proliferation and survival, but the molecular mechanisms involved in these effects are not well understood.

One of the most important genes up-regulated by vitamin D is the thioredoxin interacting protein (TXNIP), initially named as vitamin D up-regulated protein-1, VDUP-1^14^. TXNIP is an α-arrestin known to inhibit glucose uptake by binding directly to glucose transporter GLUT-1, inducing its internalization^15^. TXNIP also binds to thioredoxin (TRX)^16^, linking the intermediary and primary metabolism, the redox regulation and cell cycle^17–19^. TXNIP also competes with apoptosis signal-regulating kinase 1, ASK1, for binding to TRX. Up-regulation of TXNIP, such as observed in diabetes mellitus type 2, leads to the displacement of TRX from binding to ASK1, promoting the activation of apoptosis^20, 21^. Since vitamin D is a major regulator of TXNIP, one conceivable suggestion is that vitamin D is an important regulator of cellular and redox metabolism.

Metabolomics, the study of low-molecular-weight metabolites of physiological relevance, combined with measurements of mRNA and protein contents, as well as enzymatic activities, is a powerful approach to reveal new mechanism for cellular metabolic reprogramming associated with the disease state, as described for different cancer-subtypes^22, 23^ and also for subjects infected with viruses, as recently described by our group^24^. Recently, metabolomics was proved an important tool for understanding the role of vitamin D on multiple sclerosis^25^.

In this work, we combined ^1^H Nuclear Magnetic Resonance (NMR) metabolomics with functional and molecular biochemical assays to describe the metabolic changes elicited by vitamin D in HEK293T, an embryonic proliferative cell line adapted to high-glucose concentrations. Vitamin D treatment reprogrammed the metabolism of these cells by decreasing the polyols pathway and by channeling glucose carbons to the maintenance of the cell’s reductive power and the cell proliferative phenotype, possibly protecting them from the oxidative stress promoted by high glucose concentration.

## Results

### Vitamin D treatment alters cellular metabolic profile

To understand the effect of vitamin D on metabolism we first performed ^1^H NMR exploratory metabolomics on HEK293T cellular extracts. HEK293T is an embryonic non-cancerous proliferative cell line adapted to high-glucose concentrations. We are particularly interested in the metabolic mechanisms in the presence of high-glucose concentrations and the way cells deal with oxidative stress.

Using NMR, we first mapped the consumption and fate of glucose carbons, showing that HEK293T is highly glycolytic (Supplementary Figure 1), as expected for an embryonic cell^26^. We used growth medium containing 25 mM glucose, and after 24 hours of incubation, ~10 mM glucose was consumed, remaining ~15 mM in the culture medium. 23% of ^13^C-glucose that was consumed by the cells was metabolized to ^13^C-lactate, measured in the conditioned media (~4.5 mM after 24 hs), indicating that almost a quarter of the glucose consumed followed the anaerobic glycolytic pathway. The presence of vitamin D did not change ^13^C-glucose consumption and the amount of ^13^C-lactate export by the cells.

**Figure 1:**
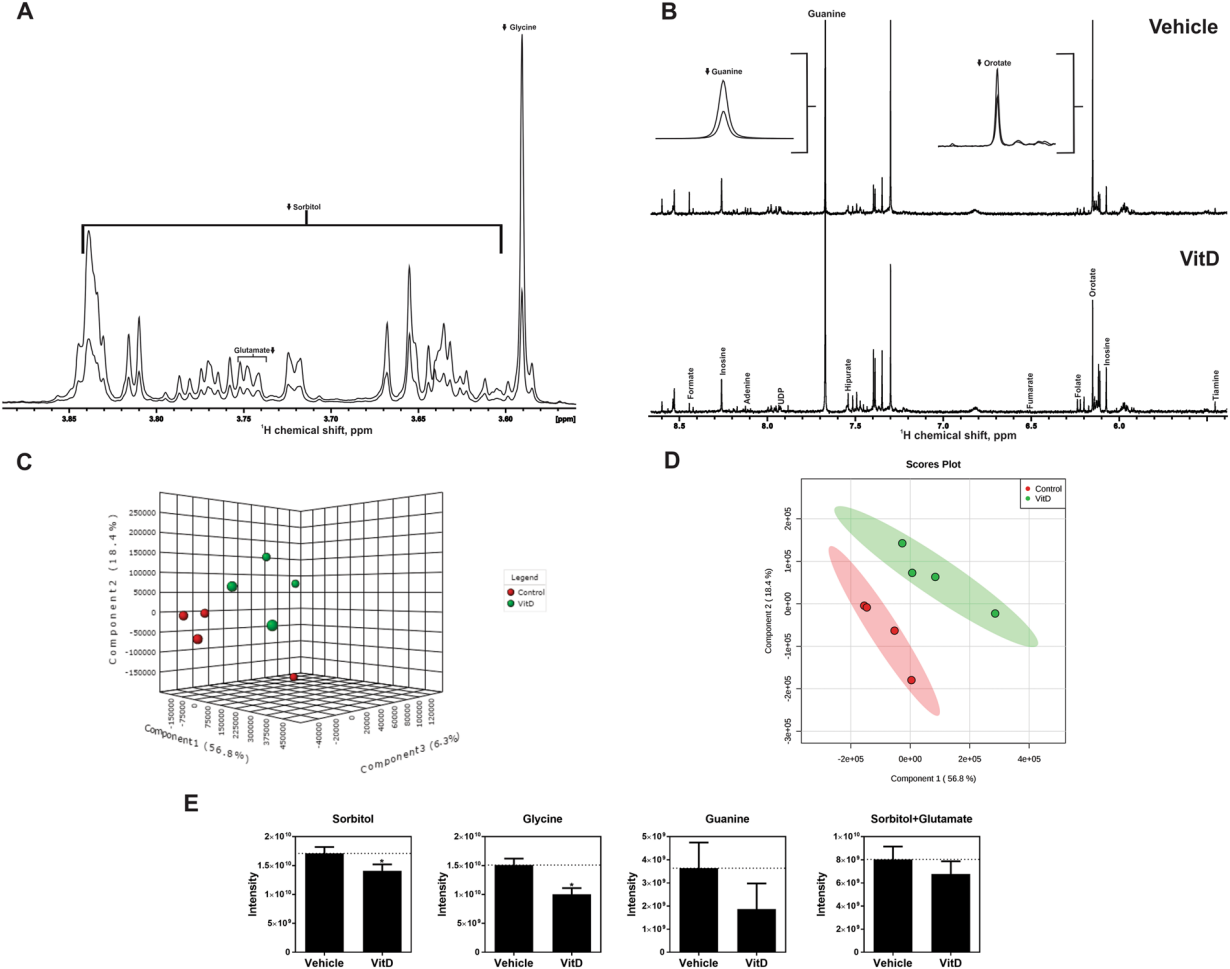
NMR metabolomics shows different metabolic profile by vitamin D treatment. 1D ^1^H NMR representative spectra from HEK293T cells polar extract of aliphatic region (**a**), and aromatic region (**b**). Arrows ↑↓ indicate increased or decreased metabolites, respectively, by vitamin D treatment. PC- Phosphocholine; GPC- Glycerophosphocholine; DSS- 4,4-dimethyl-4-silapentane-1-sulfonic acid. (**c**) 3D Score plot of Partial Least-Square Discriminant Analysis (PLS-DA), with components 1, 2 and 3. (**d**) 2D Score plot of PLS-DA with components 1 and 2. Control (vehicle) replicates (red circle) exhibit more variability than 100 nM (green circle) vitamin D treatment. (**e**) Intensity of the NMR resonance correspondent to the most significantly altered metabolites in the presence and absence of vitamin D (p value < 0.05). Values represent the average of 4 independent experiments, ± SD. For the analysis of the statistical significance with and without vitamin D we used unpaired t-test (*p value < 0.05).

NMR analysis showed that the most prevalent metabolites in HEK293T extracts are sorbitol and glycine. Figure 1A and B show a representative ^1^H NMR spectrum of the cellular extracts’ polar phase after cell treatment with vitamin D. Figure 1C and D show results of the multivariate analysis, which accounts for the HEK293T cells before and after treatment with 100 nM of vitamin D. Vitamin D reprogrammed the cells to a different metabolic phenotype, as observed by the decrease in the content of sorbitol, glutamate, glycine, orotate and guanine and small differences in the content of lactate and alanine (Fig. 1E and Supplementary Figure 2).

We observed a clear separation between the vitamin D-treated and control cells, as shown in the multivariate analysis PLS-DA score plot (Fig. 1C and D). We validated the PLS-DA analysis using cross-validation by the leave-one-out method^27–29^. We also observed a reasonable (but not strong) separation into classes in the principal component analysis (PCA), which is unsupervised and not biased toward classification (Supplementary Figure 3). It is important to note that our main emphasis was not the classification power of our model, but the hierarchy of metabolites that contributed to classification, which were remarkably similar for the PLS-DA and PCA analysis (Supplementary Figure 3).

Cross validation revealed an accuracy of 0.875, R^2^ of 0.978 and Q^2^ of 0.545 for 3 components, which is a good classification model. R2 measures how good is the fit, while Q^2^ measures the predictive ability of the model. For both parameters, the value 1 would reflect perfect fitting and predictive power^30^. Values of Q^2^ above 0.5 usually reflect a good classificatory power without overfitting. Although the classification power of the model presented here is reasonable, in this work we did not use it as a predictive tool, but rather to provide directions for the understanding of the main metabolic changes in HEK293T cells elicited by vitamin D.

**Figure 2:**
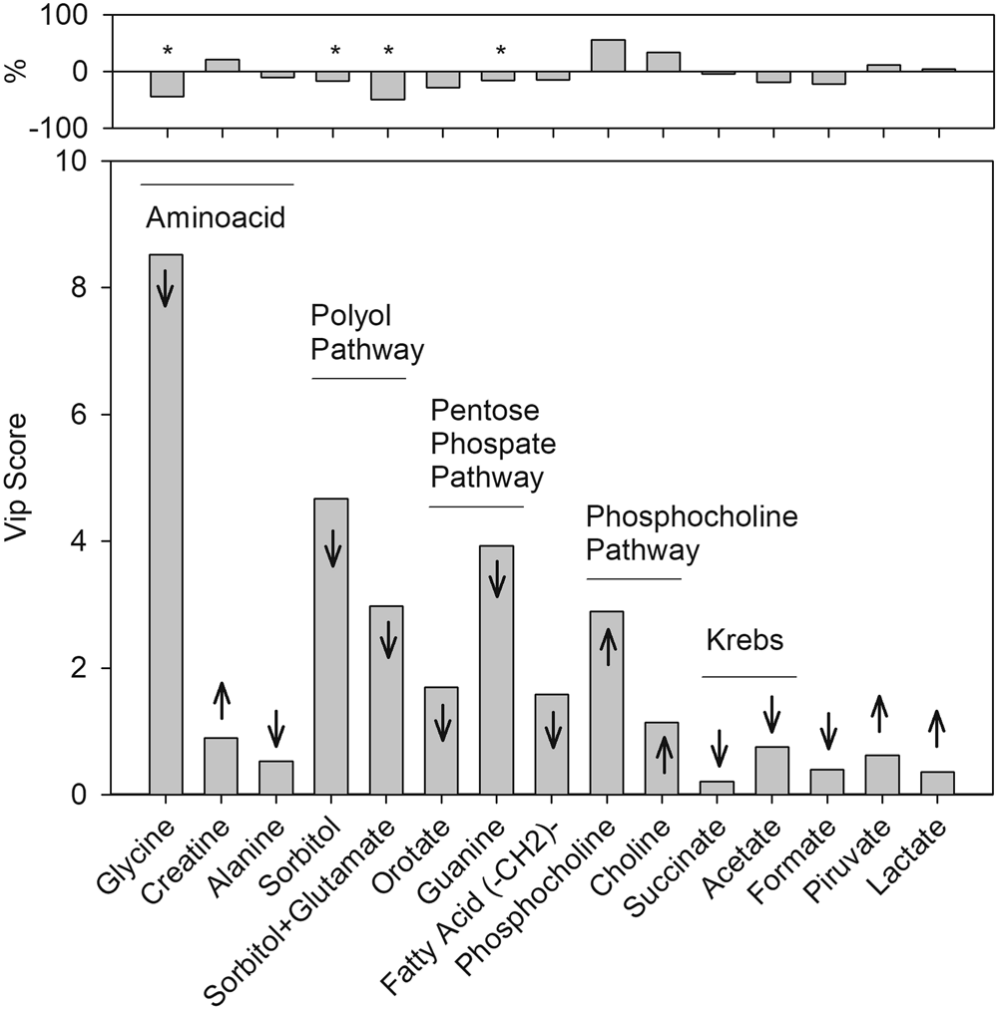
Effect of vitamin D on the metabolic profile of HEK293T cells. The figure reports the metabolites with the most discriminating power in between vitamin D treated and control HEK293T cells, according to PLS-DA multivariate and univariate analysis. The bottom graph shows the VIP score as a function of the metabolites. The arrows indicate the effect of vitamin D on the content of the metabolite (PLS-DA). The top plot shows the results of the univariate analysis, with the bars indicating the percentage of change in the average area of the peak (bucket) for each metabolite. For the analysis of the statistical significance with and without vitamin D we used unpaired t-test (*p < 0.05, Fig. 1E). Note that the univariate analysis agrees with the multivariate analysis for all metabolites. Supplementary Table 2 details PLS-DA and univariate analysis for most discriminating metabolites.

Several variables were important for group discrimination, as shown on PLS-DA loading factors on components 1, 2 and 3, and variable importance in projection (VIP) scores on PLS-DA component 1 (Supplementary Table 1). Scores on PLS-DA components 1, 2 and 3 accounted for almost 90% of the variation (Fig. 1C). From these buckets, we picked up some with the highest VIP-scores and loading factors, and added some other assigned metabolites, as shown in Supplementary Table 2. Figure 2 points to the metabolites with the most discriminating power between vitamin D treated and control HEK293T cells, according to PLS-DA multivariate and univariate statistics. The variables/metabolites with the highest VIP-scores and loading factors are shown in Fig. 2 and Supplementary Table 2.

Glycine, sorbitol, glutamate and guanine strongly contributed to the discrimination of the groups, taken by their high VIP-scores (Fig. 2, lower panel). These metabolites appear in the chemical shift values between 3.6–4.0 ppm (Fig. 1A) and presented the highest negative loading factors, indicating that vitamin D significantly decreased their content in HEK293T cells (Fig. 2, upper panel). Other amino acids and metabolites related to the pentose phosphate pathway, phospholipid metabolism, Krebs cycle and glucose metabolism also contributed to discriminating the metabolic phenotype of control and vitamin D-treated HEK293T cells (Fig. 2). These included a decrease in alanine, orotate, acetate, succinate and formate and an increase in phosphocholine, lactate, pyruvate and creatine in vitamin D-treated HEK293T cells.

To individually evaluate each metabolite, we also performed univariate analysis (Fig. 2) and confirmed the statistically significant (p < 0.05) changes of sorbitol, glycine, glutamate and guanine, as shown in Fig. 1E.

We also evaluated whether vitamin D treatment affected the cell cycle and the growth curve of HEK293T cells, but despite the metabolic effects induced by vitamin D treatment, no changes in cell proliferation were observed (Supplementary Figure 4).

### Anaerobic glycolysis is reprogrammed in the presence of vitamin D

**Figure 3:**
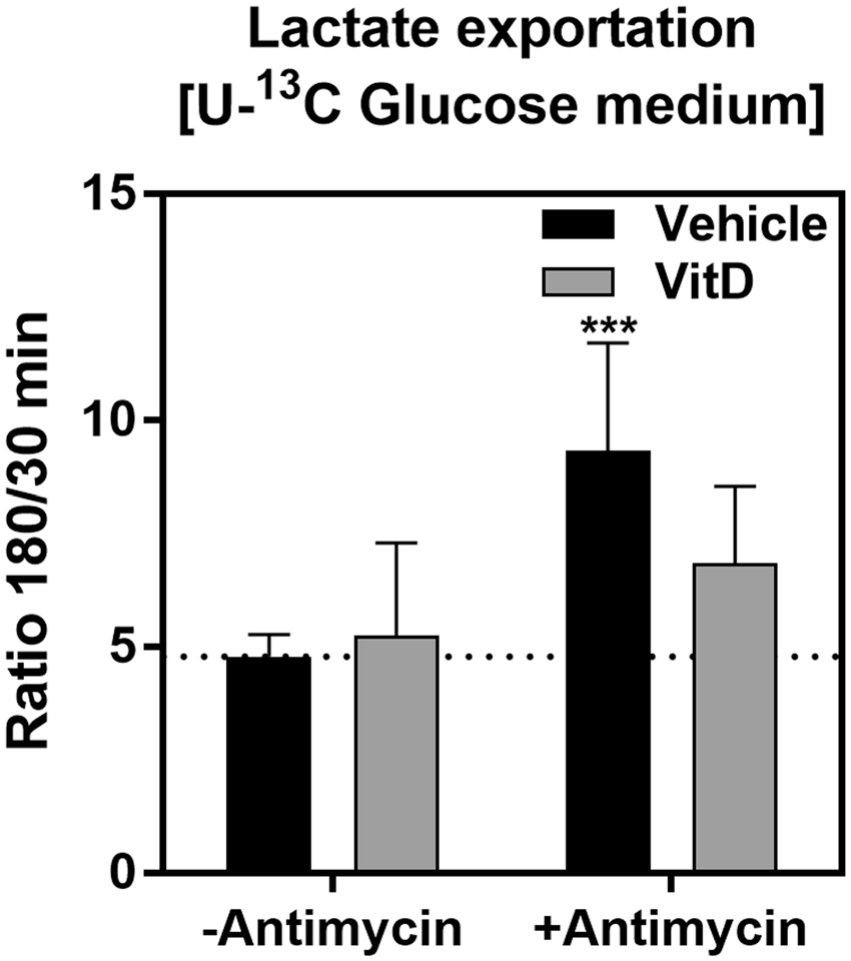
Vitamin D restrained anaerobic glycolysis. HEK293T cells were treated for 24 hours with 100 nM of 1,25 dihydroxyvitamin D3, after cell medium was changed by the same medium except ^13^C-U-glucose. Cell medium was aliquoted every 15 minutes, up to 180 minutes. After 30 minutes, antimycin A (inhibitor of mitochondrial complex III) was added. Lactate in the culture medium was measured by one-dimension ^13^C-NMR spectra acquisition, with proton decoupling, of aliquots of the culture medium. ^13^C-Lactate accumulation was calculated by the ratio of peak intensities in 180 min:30 min. We measured the lactate peak intensity for every 15 min. The intensity ratio 180/30 min normalizes for small changes at the beginning of the kinetics (30 min) and better reports for the significance of the effect of vitamin D and antimycin. Only after 120 min (we chose 180 min) the difference among the tested condition arose. Values represent the average of 3 independent experiments, ± SD. For the analysis of the statistical significance with and without vitamin D we used unpaired t-test (*p < 0.05).

We observed a slight increase in intracellular lactate concentration in vitamin D-treated cells (Fig. 2, Table S2), corroborating the subtle effect of vitamin D on glycolysis, which was already described for other cell models^10, 11, 31^. To better understand this effect, we measured the fate of glucose carbons by incubating HEK293T cells with ^13^C-glucose and measuring ^13^C-lactate accumulation in the culture medium. Both ^13^C-glucose consumption and ^13^C-lactate accumulation in the culture medium were not affected by vitamin D, in agreement with the subtle increase in intracellular concentration of lactate (Fig. 2, Table S2). This experiment agrees with the ^13^C-lactate accumulation observed in Supplementary Figure 1. Remarkably, when HEK293T cells were exposed to antimycin A, an inhibitor of cellular respiration, which forces cells to rely solely on anaerobic glycolysis, vitamin D-treated cells exhibited a less pronounced increase in ^13^C-glucose consumption when compared to control cells, which displayed a 2-fold increase in lactate accumulation (Fig. 3). These results support that anaerobic glycolysis is reprogrammed in the presence of vitamin D. It could be suggested that (1) vitamin D impairs glycolytic capacity of HEK293T cells; or (2) the small increase in intracellular lactate observed in PLS-DA is due to a decreased transport out of cell. These hypotheses were tested by different approaches, shown in the next topics.

### Vitamin D induced changes in the activity of glycolytic enzymes

**Figure 4:**
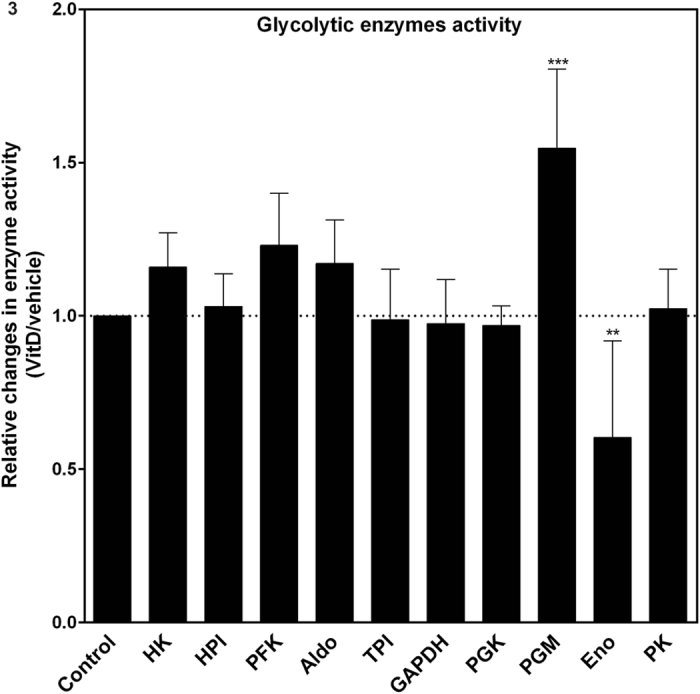
Vitamin D treatment induces a decrease in enolase and an increase in PGM activities. To perform enzymatic assays, HEK293T cells were treated for 24 hours with 100 nM of vitamin D or vehicle before cell lysis. The activity of glycolytic enzymes was assayed as described in methods. The graph indicates the fold change on the activity of each enzyme after vitamin D-treatment in relation to vehicle treatment (control). Values represent average ± SD; n = 6 for HK, n = 4 for PGM, n = 7 for Eno and n = 3 for the other enzymes. HK = hexokinase; HPI = hexose phosphate isomerase; PFK = phosphofructokinase; ALDO = aldolase; TPI = triose phosphate isomerase; GAPDH = glyceraldehyde 3-phosphate dehydrogenase; PGK = phosphoglycerate kinase; PGM = phosphoglycerate mutase; Eno = enolase; PK = pyruvate kinase. For the analysis of the statistical significance with and without vitamin D we used unpaired t-test (**, ***p < 0.05).

To better evaluate vitamin D effects on glycolysis, we measured the activity of all 10 glycolytic enzymes (Fig. 4). Phosphoglycerate mutase (PGM) activity presented a 50% increase and enolase activity a 45% decrease in cells treated with vitamin D when compared to controls. The activity of enolase decreased from 42 ± 9 to 24 ± 16, while phosphoglycerate mutase increased from 299 ± 52 to 452 ± 61 (nmol of product × min^−1^ × µg protein^−1^), for control and vitamin D-treated cells, respectively. These results reinforce the idea that vitamin D reprogram glucose metabolism. We did not observe significant changes in the activity of the other glycolytic enzymes.

### Vitamin D does not alter mitochondrial respiration

**Figure 5:**
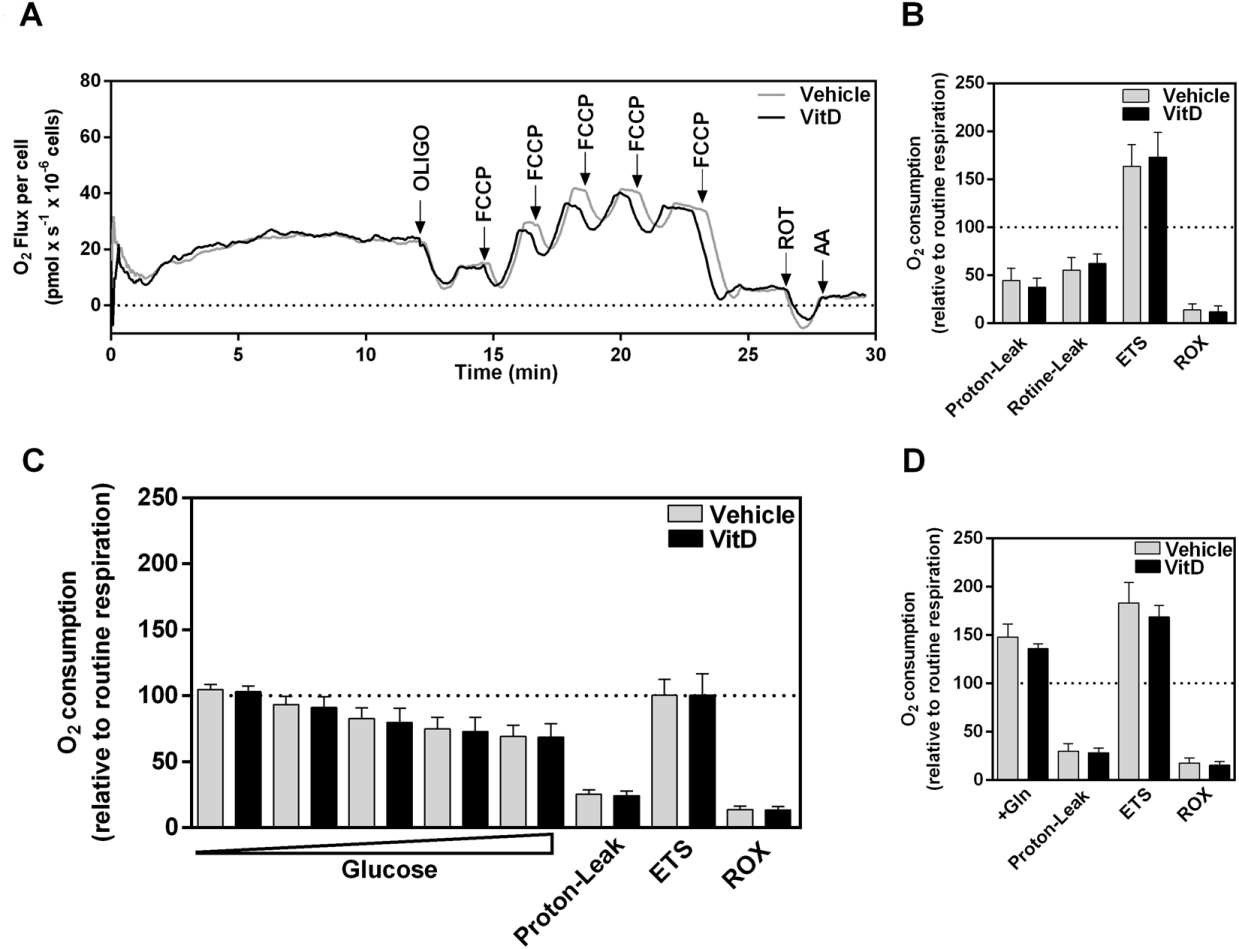
Vitamin D does not affect mitochondrial respiration. High-resolution respirometry assay using Oroboros 2 K. Oxygen consumption was evaluated after the addition of modulators of mitochondrial activity. Basal respiration corresponds to the oxygen consumption of resting cells. By treating the cells with oligomycin (1 mg/mL), an inhibitor of ATP synthase, we obtained the oxygen consumption related to the proton leak through the inner mitochondrial membrane. Treatment with the protonophore FCCP, titrated to increase its concentration in 50 nM after each addition) allowed the measurement of the maximum oxygen consumption, reflecting the maximum capacity of the electron transport system. Addition of rotenone (50 nM) and antimycin A (1 mg/mL), inhibitors of complex I and III, respectively, revealed the oxygen consumption not associated with mitochondrial activity, the residual respiration. Representative traces of oxygen consumption rate in intact cells (**A**) and average results of O_2_ consumption relative to the basal respiration (**B**), or after adding either glucose (**C**), or glutamine (**D**). Bars represent mean ± SD of six independent experiments.

To understand the contribution of oxidative metabolism to HEK293T cells metabolic phenotype and the effect of vitamin D on HEK293T respiratory activity, we performed a set of experiments using high-resolution respirometry. Figure 5A shows a representative trace of oxygen consumption rate in intact cells, respiring in a complete culture medium, containing glucose, glutamine and other respiratory substrates. Figure 5B presents the average effect of vitamin D on mitochondrial function parameters, including proton leak, maximal respiration capacity and residual oxygen consumption rate, all measurements expressed as percentage of the basal respiration. Oxygen consumption rate in the presence of oligomycin, a compound that inhibits ATP synthase activity, is referred to as “leak” respiration since it evaluates oxygen consumption uncoupled to ATP synthesis, while the fraction of oxygen consumption that was inhibited by oligomycin represents the “coupled” respiration. Maximal oxygen consumption rates were evaluated in the presence of p-triflouromethoxyphenylhydrazone (FCCP), which is a measure of electron transfer system (ETS) capacity, and non-mitochondrial or residual oxygen consumption rate (ROX) was measured in the presence of antimycin A. All these respiratory parameters were not affected by vitamin D incubation.

To investigate possible effects of vitamin D on the cell preference for specific respiratory substrates, we evaluated mitochondrial respiration in the presence of glucose (Fig. 5C) or glutamine (Fig. 5D) alone. Glucose induced a concentration-dependent decrease in cellular respiration, with no significant differences in vitamin D-treated and control cells. The decrease in respiration caused by glucose is compatible with the Crabtree effect^32^, showing that HEK293T cells present a proliferative phenotype.

Since vitamin D caused a decrease in glutamate content in HEK293T cells, we next sought to investigate cellular respiration in the presence of glutamine as the sole respiratory substrate. Addition of glutamine increased cellular respiration by approximately 50%, with no significant differences upon vitamin D incubation. Collectively, the results shown in Fig. 5 indicate that mitochondrial bioenergetics of HEK293T cells was not affected by vitamin D and that the alteration in the metabolic profile is not directly related to alterations in mitochondrial oxidative pathway.

### Key genes involved on glucose metabolism were modulated by vitamin D

**Figure 6:**
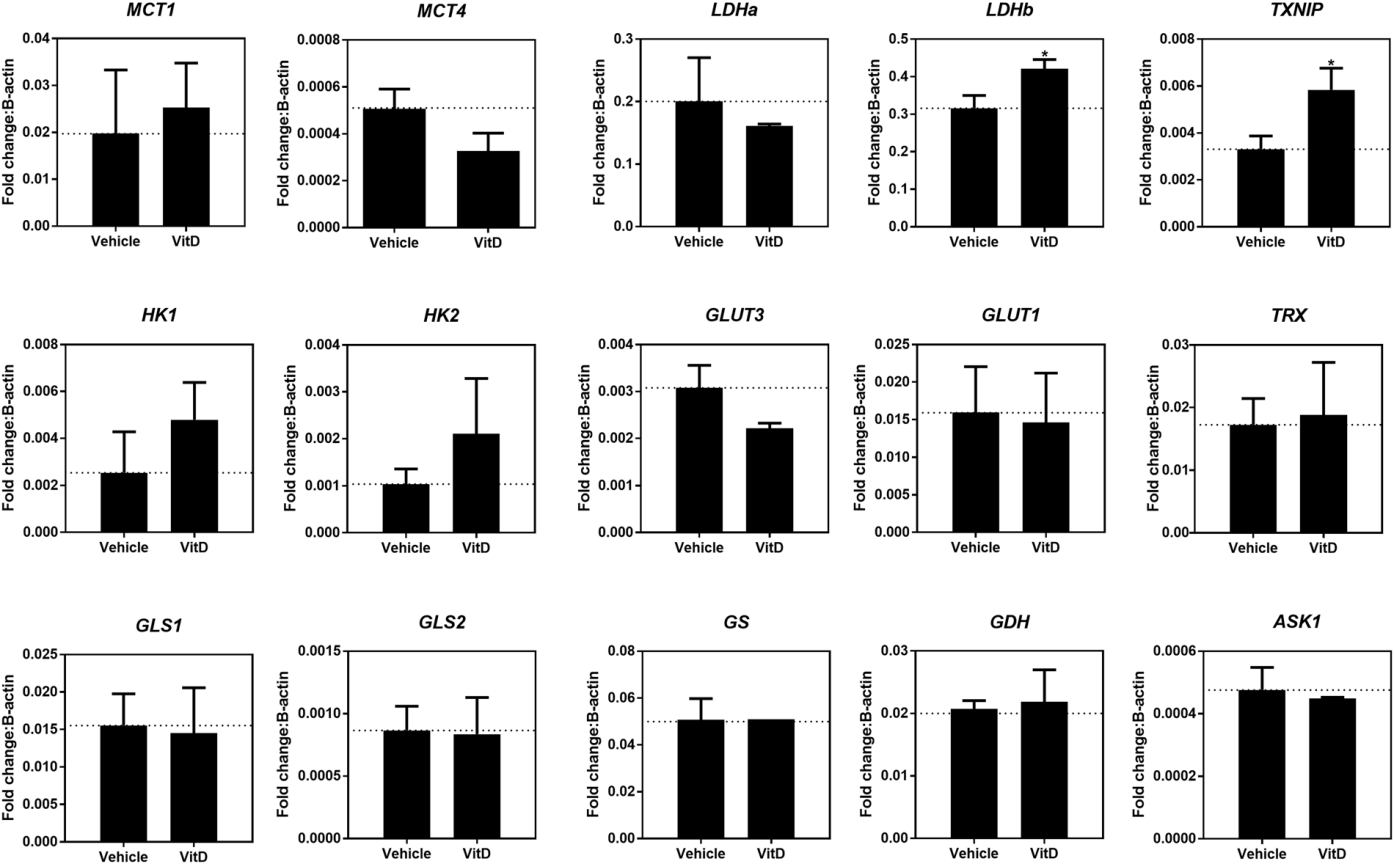
Vitamin D modulate expression of important genes. HEK293T cells were treated for 24 hours with 100 nM vitamin D, and total RNA were extracted followed by RT-qPCR. *TXNIP* and *TRX*, are linked to oxidative stress. *ASK1* is linked to apoptose. *MCT1/4* are linked to lactate exportation. *GLUT1/3* are linked to glucose transport. *HK1/2* and *LDHa/b* are linked to glycolysis. *GLS1/2*, *GS* and *GDH* are linked to glutamine metabolism. Each bar corresponds to the triplicate mean fold change relative to β-actin gene. Error bars correspond to SD of biological triplicate. Statistical significance was evaluated using unpaired t-test (*p < 0.05) according Material and Methods.

The functional experiments presented on the previous sections were followed by mRNA quantification for several genes linked to glucose and glutamine metabolism, redox regulation and apoptosis (Fig. 6). We chose to evaluate the transcription of the following genes: glucose transporters 1 and 3 (GLUT 1/3); hexokinase 1 and 2 (HK 1/2), which catalyze the first step of the glycolytic pathway; lactate dehydrogenase (LDH), which reduces pyruvate to lactate (LDHa) and the reverse reaction (LDHb); monocarboxylate transporters 1 and 4 (MCTs1/4), which act on lactate transport into and out to the cells; TRX; apoptosis signal-regulating kinase 1 (ASK1), which are linked to apoptosis; glutamine synthetase (GS), which synthesizes glutamine from glutamate; glutaminase 1 and 2 (GLS1/2), which breaks down glutamine to glutamate; and glutamate dehydrogenase (GDH), the enzyme that converts glutamate into α-ketoglutarate. As a control of vitamin D effect on HEK293T cells, we confirmed the increase in TXNIP gene transcription (Fig. 6) and protein expression by vitamin D (Supplementary Figure 5).

*LDHa* and *MCT4* transcripts, related to lactate production and efflux from the cells, were downregulated, and *HK1*, *HK2* and *LDHb* were upregulated by vitamin D incubation.

Glucose transporter GLUT1 is the most abundant isoform of the glucose transporters and is ubiquitously expressed in most cells^33^, and can be down-regulated by TXNIP^15^. In the present study, despite TXNIP overexpression (Supplementary Figure 5), GLUT1 mRNA content did not show any difference by vitamin D incubation (Fig. 6). Moreover, no difference in glucose consumption was observed after 24 hours of U-^13^C-glucose addition, as shown in Fig. 4. Together, these data indicate that vitamin D-induced TXNIP up-regulation was not sufficient to reduce neither GLUT1 transcript nor glucose uptake by HEK293T cells.

GLUT3 was originally assigned to be the neuronal GLUT^34^, but more recently, several studies described GLUT3 as present in different cell lines^35–37^. GLUT 3 transcript levels were decreased in HEK293T cells by vitamin D incubation (Fig. 6), suggesting that vitamin D effect on glucose transport via TXNIP up-regulation, at least in these cells, might involve more complex mechanisms.

## Discussion

**Figure 7:**
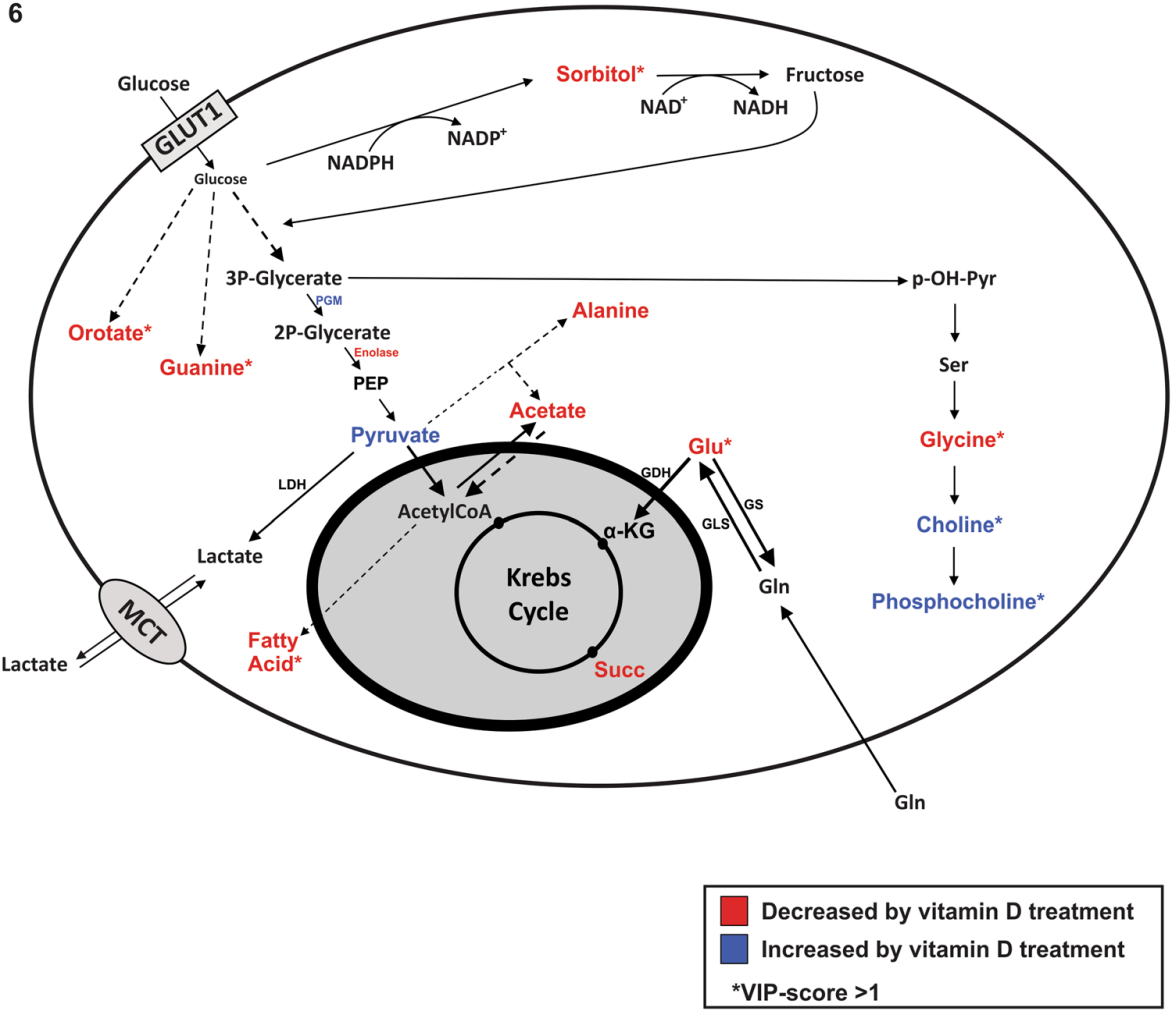
HEK293T cells has several metabolic pathways affected by vitamin D. Blue and red colors represent increased and decreased metabolites, respectively, by vitamin D treatment. (↑) Genes or enzymes up-regulated by vitamin D; (↓) genes or enzymes down-regulated by vitamin D. (*) Metabolites with VIP-score on PLS-DA component 1 higher than 1.

In the present study, we showed several evidences that vitamin D induced metabolic changes in HEK293T cells exposed to high glucose concentrations, mimicking hyperglycemic glucose concentrations. Vitamin D reprogrammed the metabolism of HEK293T cells, protecting them from oxidative stress and helping to maintain their highly proliferative phonotype. This proposal is based in the following metabolic consequences related to the cellular treatment with vitamin D: (a) the decrease in the concentration of sorbitol; (b) the decrease in glycine, glutamate and alanine intracellular contents, together with an increase in pyruvate, choline and phosphocholine; (c) the decrease in guanine and orotate concentration and (d) a subtle reprogramming of glycolysis evidenced by inhibition of enolase and activation of phosphoglycerate mutase (PGM) activity in the extract. Each of these evidences is explored in detail as follows. Figure 7 summarizes the metabolic reprogramming caused by vitamin D.

It is well stablished in the literature that the exposure of cells to high glucose concentrations elicits a series of events, which, when persistent, causes pathological conditions to the organism. One of the main consequences of high glucose exposure is the activation of the polyol pathway^38–40^, which, in the non-disease context, is known to be important to cellular osmoregulation^41^, but in the context of diabetes it is associated to tissue damaging during hyperglycemia^40, 42^. The activation of the polyol pathway by high glucose levels (hyperglycemia) is correlated to increases in intracellular ROS production, which may account for some of the complications of diabetes, such as atherosclerosis and cardiomyopathy. Cells of diabetic patients display increased sorbitol levels, which is associated to an increased production of intracellular ROS, triggering oxidative stress^40^. The polyol pathway is unique in the capacity of interchanging NADPH with NADH, decreasing the reductive power of the cells, and this is the most accepted hypothesis to explain the polyol pathway-dependent oxidative stress in a hyperglycemia situation. In this context, the clinical correlation between vitamin D deficiency and the development of diabetes mellitus type 2^43^ suggests a protective role of vitamin D from the harmful effects of hyperglycemia.

Indeed, in the present study, the most noticeable metabolic feature of HEK293T cells was that its most prevalent metabolite is sorbitol. Sorbitol is generated in the polyol pathway, as depicted in Fig. 7. In our experimental model, HEK293T cells were exposed to high glucose concentrations (25 mM), mimicking hyperglycemia, which could explain the high intracellular levels of sorbitol we found in this condition. Following this observation, we showed that vitamin D treatment significantly reduced the intracellular concentration of sorbitol, which presumably prevent cells from oxidative stress.

We observed a significant decrease in the glycine content in cells treated with vitamin D. Glycine is primarily being used in choline and phosphocholine biosynthesis, which is increased in the presence of vitamin D. The products of choline phospholipid metabolism, such as phosphocholine, diacylglycerol and phosphatidic acid, are second messengers that are essential for mitogenic activity of growth factors, participating in the activation of the MAP/ERK pathway (ras-raf-1-MAPK cascade) and protein kinase C pathway^44^. The activation of this pathway is particularly important for highly proliferative cells, such as HEK293T.

The significant decrease in guanine content in vitamin D-treated cells suggests an increased utilization of purine molecules, as glycine contributes with up to four of the five carbons atoms in the purine ring, including indirectly contribution via N10-formyltetrahydrofolate^45^. Additionally, the lower content of orotate upon vitamin D treatment (Figs 1C and 2) also points to an increased utilization of pyrimidine molecules, as orotate is an intermediary in pyrimidine synthesis.

We also observed a decrease in glutamate upon treatment with vitamin D, which was not related to changes in the glutamine metabolism, since no differences were observed in the respirometric parameters when glutamine was the only substrate given (Fig. 5). Additionally, both mRNA contents for glutamine synthase and glutaminase were like those found in control cells (Fig. 7) and glutaminase activity was not altered upon vitamin D incubation (Supplementary Figure 6). Incorporation of ^15^NH_4_ into glutamate after cell incubation with ^15^NH_4_Cl did not differ between vitamin D-treated and untreated cells (data not shown).

The polyol pathways, glycine metabolism and purine and pyrimidine pathways are all connected in some way to glycolysis (Fig. 7). We demonstrated that the relative importance of glycolysis is high in HEK293T, since ~23% of the glucose that entered the cells were converted and exported as ^13^C-lactate. Vitamin D elicited a subtle reprogramming of glycolysis. We observed lower enolase activity and higher phosphoglycerate mutase (PGM) activies upon vitamin D treatment. Glycolysis was neither inhibited or activated by vitamin D (glucose consumption and lactate accumulation is unaltered). Rather, it is subtly modulated in these two sequential enzymatic reactions. When a single enzyme is downregulated, the velocity of a pathway can be deeply interfered, resulting in the reduction or accumulation of metabolites that can take other metabolic fate. It has been shown that the inhibition of enolase alone elicited a series of biological effects related to glucose homeostasis^46^. Indeed, enolase and PGM catalyze sequential glycolytic reactions. Therefore, the decrease in enolase activity might favor the reverse reaction of PGM, which would suggest a flux modulation of glycolytic intermediates by Vitamin D.

Lactate efflux was similar in the presence and absence of vitamin D, unless antimycin A was present. This result implies that, under conditions where oxidative/mitochondrial metabolism is not impaired, the alterations in glycolysis induced by vitamin D does not affect energy metabolism and, in fact, might guarantee the deviation of glucose carbons to other pathways. On the other hand, when mitochondrial respiration was inhibited by antimycin A, lactate production did not increase in the same manner as in control cells. This difference might be explained by the decrease in enolase activity induced by vitamin D. Additionally, one would suggest that the decrease in MCT4 expression in vitamin D-treated cells (Fig. 6) might have impaired lactate efflux when cells were exposed to antimycin A.

In this study, HEK293T, an immortalized non-tumorigenic cell line, was treated with vitamin D for 24 hours^47^. An important outcome of the present research is the small and undetected participation of TXNIP in the effect of vitamin D on HEK293T cells. The lack of effect on cell proliferation, apoptosis and growth arrest-related pathways, reinforced by the results of ASK-1 mRNA expression, might indicate that vitamin D effects on non-tumorigenic cells seems to be related to cellular metabolism remodeling. In contrast, in cancer cell models, up-regulation of TXNIP led cells to apoptosis and growth arrest^48–50^.

NMR-based metabolomics is limited to detection of the metabolites that are in higher concentrations. We could not detect the variations of important metabolites, such as NADH/NAD^+^, NADPH/NADP^+^ and glutathione. Glutathione concentrations in cells are usually detectable by ^1^H NMR (mM range), but we could not detect in the cell extracts of HEK293T because its main resonance (3.77 ppm) is overlapped by the sorbitol resonances, which is in great excess.

Taken together, the observed effects of vitamin D regulated redox homeostasis (protection against oxidative stress), supported by the decrease in sorbitol concentration, and helped maintaining the highly proliferative phenotype, supported by the decrease in glycine and guanine and orotate concentration and increase in choline and phosphocholine concentration. Additionally, the decrease in orotate and guanine are an indication of the increased biosynthesis of purine and pyrimidines. The groundwork established by our metabolic findings suggests that mitogenic efficiency and bursting the reductive power by rearranging glucose carbons between the glycolytic and the polyols pathways are important features of vitamin D action in proliferative cells, as is the case of HEK293 T cells. Since recent reports correlate vitamin D deficiency to the development of type 2 diabetes mellitus^43^, this study may contribute to the understanding of the metabolic effect of vitamin D upon hyperglycemia, suggesting a possible role in protecting the cells against the oxidative stress. Further studies, analyzing the glutathione, NAD(P)^+^/NAD(P)H levels will be interesting to support the findings of the present work.

## Materials and Methods

### Cell Culture

HEK293T cells were cultured in DMEM-High glucose medium with 1X Penicilin/Streptomicin and 10% fetal bovine serum at 37 °C in a humidified atmosphere at 5% CO2. Cells were tested for mycoplasma by PCR. Cells were treated for 24 hours with 1 nM and 100 nM of 1.25 dihydroxyvitamin D3 (Sigma^®^) solubilized in 95% ethanol. For growth curves, 1–2 × 10^3^ cells/cm^2^ were seeded in complete medium with the respective additions of drugs and/or reagents, which was renewed every other day. Dishes were periodically harvested and cells were detached, fixed in formaldehyde 3.7%, diluted in Phosphate Buffered Solution (PBS) and stored to be later counted at Muse® Cell Analyser (Millipore).

### Cell cycle analysis by flow cytometry

Cells were fixed in ethanol 70% before total DNA staining with propidium iodide at 50 µg/ml. RNA were digested with RNAseA at 100 µg/ml for 20 minutes at room temperature. Samples were submitted to flow cytometry at BD FACSCan (BD Biosciences - Franklin Lakes, New Jersey, EUA). Analyses were performed using Flowing software (version 2.5.1), where marks correspondent to the cells with DNA content of G1, S and G2 were created in histogram plots to determine the number of events in each cell cycle phase.

### NMR Metabolomics

Supplementary Figure 7 describes the experimental design used for the experiments describing NMR metabolomics (Fig. 1, Supplementary Figures 2 and 3) and to measure the fate of glucose in the cell (Fig. 3 and supplementary Figure 1). Cells were thawed and grown in high-glucose DMEM for two passages. 1 × 10^6^ cells were plated in 100 mm petri dishes and grown for 24 hs to approximately 60% of confluency. At this point we added 100 nM of vitamin D. The cells were grow for another 24 hs in the presence (and absence) of vitamin D, reaching approximately 90% of confluency. We then pelleted the cells for metabolite extraction.

Our protocol for metabolite extraction was adapted from Bligh and Dyer biphasic extraction^51^. 3 × 10^6^ cells (~30 mg) were extracted with one step of methanol/chloroform/water (2:1:0.8, 2.4 mL:1.2 mL:1.5 mL, respectively), vortexed for 2 minutes after each solvent addition and followed by shaking for 30 minutes on ice. The mixture is finally centrifuged at 4600xg for 20 minutes at 15 °C. Supernatant (polar aqueous phase) was dried on SpeedVac, and kept at −80 °C. Dried extracts were suspended in 50 mM phosphate buffer, pH 7.4, 10% of D_2_O and 0.1 mM of 4,4-dimethyl-4-silapentane-1-sulfonic acid (DSS) for reference.

NMR spectra were collected in Bruker Avance IIIHD operating at 500.13 MHz for ^1^H at 298 K. ^1^H spectra were acquired with excitation sculpting for water saturation, 12.9836 ppm spectral width, 1.74 s relaxation delay, 32 K points and 3 K accumulations. ^1^H-^13^C HSQC, ^1^H-^1^H TOCSY, and pJRES spectra were used for assignments. Spectra were processed in TOPSPIN 3.2 (Bruker-Biospin), and exported to AMIX (Bruker-Biospin).

For statistical sampling, we collected datasets for 4 independent polar extracts for vitamin-D treated cells and 4 for vehicles. Univariate analysis was done by multiple t test, considering same SD between control and vitamin D treatment. Analysis was done on GraphPad Prism 6.0, with confidence level of 95%. For multivariate analysis, spectra were calibrated using 4,4-dimethyl-4-silapentane-1-sulfonic acid (DSS) as internal reference. The spectra showed a high prevalence of two metabolites (sorbitol and glycine). We used Pareto scaling to reduce the impact of sorbitol/glycine in the statistical analysis and keep the data structure intact. The spectra were binned at 0.02 ppm followed the deletion of water and DSS signals.

We used statistical multivariate methods to discriminate the effect of vitamin D on the cell metabolism. We used Principal Component Analysis (PCA) and Partial Least Square- Discriminant Analysis (PLS-DA). To validate class discrimination and avoid overfitting, we validated the PLS-DA using permutation test (1000 permutations) based on separation distance, B/W-ratio and cross-validation by leave-one-out method^28, 29, 52^.

To rank the relative importance of each metabolite we calculated the variable importance in the projection (VIP-score, Fig. 2, Table S2) and the PLS-regression coefficients for components 1, 2 and 3 (comp1, comp2 and comp3, Fig. 2, Table S2). VIP scores reflects the weighted sum of squares of the PLS loadings and it weights the amount of Y-variance in each dimension. The regression coefficients reflect the weights as a function of the reduction of the sums of the squares across the number of PLS components. We used MetaboAnalyst 3.0 for all multivariate analysis and PLS-DA validation^27, 53^.

### Western Blot

Whole cell lysates were made from 10^6^ cells, with buffer containing 10 mM tris-HCl pH 7.0, 0.25 M sucrose, 20 mM NaF, 1 mM DTT, 5 mM EDTA, and 1X PIC (protease inhibitor cocktail). Total protein was measured by Bradford assay method. SDS-PAGE were done with 50 µg of protein, and transferred to nitrocellulose membrane (iBlot -#IB1001-life technologies). Total protein profiles were visualized by Ponceau and TXNIP were measured by anti-TXNIP antibody (#40–4600-life technologies), and b-actin were used as loading control (#A5316), with WesternBreeze (#WB7103-life technologies). Densitometries were done at ImageJ 1.6.0. Western blots were done in biological triplicate.

### RT-qPCR

Total RNA was extracted from 5 × 105 cells, by RNeasy Kit (Qiagen). The RNA quality and quantification was checked on Nanodrop (Thermo). Reverse Transcriptase (RT) reactions were done on a PTC-100 thermocycler, with 1 µg of total RNA by two-step RT-qPCR kit (Promega^®^). qPCRs were done at StepOnePlus (Applied Biosystems^®^), 1X of Master-Mix (Applied Biosystems^®^), and 1 μM of each primer pair (Forward + Reverse), for genes (5′-3′):

*TXNIP:* F-TCGGCTTTGAGCTTCCTCAG; R-AGCAGACACAGGTGCCATTA,

*TRX:* F-GACGCTGCAGGTGATAAAC; R-CTGACAGTCATCCACATCTAC,

*GLUT1:* F-AATGCTGATGATGAACCTGCT, R-CAGTACACACCGATGATGAAG

*GLUT3:* F-CTTTCTCATCCCACGCACTC; R-CACTCGGTCTCTCCTAAGCA

*MCT1:* F-GTGGCTCAGCTCCGTATTGT*;* R-GGACAGGACAGCATTCCACA

*MCT4*, F-AGGCAAACTCCTGGATGCGAC; R-GGCTCTTTGGGCTTCTTCCT

*LDHA:* F-ACCACTGCCAATGCTGTA; R-CAGGATGTGACTCACTGG

*LDHB:* F-GTGGTTTCCAACCCAGTGGAC; R-CAGCCATAAGGTAGCGAAATC

*HK1:* F-GATCATCGGCACTGGCACCAA; R-CCAAAGGCTCCCCATTCTGTA

*HK2*: F-ATGAGGGGCGGATGTGTATCA; R-GGTTCAGTGAGCCCATGTCAA

*ASK1: F-*AAGTCCCAACCCATAGAAATTCCT, R-AGCCAGTCGGTAAGTTCAGAATCTT

GS: F-CCTGCTTGTATGCTGGAGTC; R-GATCTCCCATGCTGATTCCT

GDH: F-CCAGTAGCAGAGATGCGTCCA; R-CCAGACATGAGCACAGGTGAG

GLS1: F-AGAACCGGTCGCGGCAATCCTAGCG; R-CTGAGGCCACCAGCTCTTTGCCCTCG

GLS2: F-GCAGAGAGAAGACGCCACACAG; R-GCATCTCGCTCATGCAGTCT

*B-actin:* F-TTCCTTCCTGGGCATGGAGTC, R-AGACAGCACTGTGTTGGCGTA

SYBR-Green method was used with master mix (Applied Biosystems^®^). Gene expression were normalized by B-actin gene with ΔCT method.

### Oxygen consumption of intact HEK293T cells and calculation of respiratory parameters

Oxygen consumption rates were measured by high resolution respirometry (Oroboros Oxygraph-2k) in intact HEK293T cells after treatment with 100 nM Vitamin D or vehicle, for 24 hours. Cells (10^6^ cells/mL) were suspended in the culture medium (DMEM, 25 mM glucose) without fetal bovine serum. Cells were added to the respiration chamber and the basal respiration was measured. Subsequently, oligomycin (1 mg/mL, final concentration) was added to record non-coupled respiration, referred as “LEAK”. The oxygen consumption that is insensitive to oligomycin represents the respiration related to both proton leak through the inner mitochondrial membrane and the non-mitochondrial oxygen consumption. Conversely, oxygen consumption rate that is inhibited by oligomycin corresponds to the respiration coupled to ATP synthesis^54^. The maximum uncoupled respiration was measured in the presence of optimum carbonyl cyanide p-(trifluoromethoxy) phenylhydrazone (FCCP) concentration, after titration. Maximal uncoupled respiration is a measure of the electron transport system capacity, and is referred as “ETS”. Rotenone (50 nM, final concentration) and antimycin A (1 mg/mL, final concentration), inhibitors of complex I and complex III, were used to evaluate the non-mitochondrial respiration, or the residual oxygen consumption, referred as “ROX”. To evaluate the effect of glutamine or different glucose concentration on HEK293T cells respiration, cells were suspended in DMEM without glucose, glutamine and pyruvate, prior to the addition of oligomycin, 2 mM glutamine or 0.5–5 mM glucose to the chamber. Data acquisition and analysis were done with DatLab 4.3 software (Oroboros Instruments, Innsbruck, Austria).

### Glycolysis Enzymatic Assays

Enzymatic assays were adapted from previously described procedures^55, 56^ For sample preparation, cells were resuspended in 10 mM Tris:HCl, 0.25 M sucrose, 20 nM NaF, 1 mM sodium orthovanadate, 5 mM EDTA, 1 mM DTT (pH 7.0) and lysed with freeze-and-thaw in liquid nitrogen. Cell debris were removed by centrifugation at 200 × g for 5 minutes and the supernatant was used for enzymatic assays with coupled enzymatic systems, in which the measurement of NADH production/degradation is performed by measuring the absorbance of a reaction media after the addition of the sample (cell lysate) at 340 nm. Reaction medium without the cell lysate was constant over time and used as blank. After the addition of the sample to the reaction medium, the increase or decrease of the absorbance at 360 nm over time was used to calculate the amount of product formed. The reaction medium for each enzyme contained: (1) for hexokinase (HK): 50 mM Tris-HCl pH 7.4, 5 mM MgCl2, 2 mM sodium azide, 1 U/ml G6PDH, 0,5 mM β-NAD, 0,1% triton-X, 10 mM ATP and 1–5 mM glucose; (2) for hexose phosphate isomerase (HPI): 100 mM Tris-HCl pH 7.4, 1 U/ml G6PDH, 0,5 mM β-NAD and 1 mM frutose-6-phosphate; (3) for phosphofrutokinase (PFK): 50 mM Tris:HCl pH 7.4, 5 mM MgCl2, 5 mM (NH4)SO4, 1.5 U/ml aldolase, 3.2 U/ml; (4) for triosephosphate isomerase, 3.2 U/ml glycerophosphate dehydrogenase, 0.1 mM ATP, 0.25 mM β-NADH, 1 mM frutose-6-phosphate; (5) for aldolase: 100 mM Tris-HCl pH 7.4, 3.2 U/ml triosephosphate isomerase, 1 U/ml glycerophosphate dehydrogenase, 0.25 mM β-NADH, 5 mM fructose-1,6- bisphosphate; (6) for triose phosphate isomerase (TPI): 100 mM Tris-HCl pH 7.4, 2 U/ml glycerophosphate dehydrogenase, 0.25 mM β-NADH, 1 mM glyceraldehyde-3- phosphate; (7) for glyceraldehyde 3-phosphate dehydrogenase (GAPDH): 100 mM Tris-HCl pH 7.4, 2 mM MgCl2, 1 mM ATP, 1 mM EDTA, 13 U/ml phosphoglycerate kinase, 0.25 mM β-NADH, 5 mM 3-phosphoglycerate; (8) for phosphoglycerate kinase (PGK): 50 mM Tris-HCl pH 7.4, 2 mM MgCl2, 1 mM ATP, 1 mM EDTA, 5 U/ml gliceraldehyde-3-phosphate dehydrogenase, 0.25 mM β-NADH, 5 mM 3-phosphoglycerate; 24 (9) for phosphoglycerate mutase (PGM): 100 mM Tris-HCl pH 7.4, 5 mM MgCl2, 3 mM ADP, 1 mM EDTA, 4 U/ml pyruvate kinase, 8 U/ml lactate dehydrogenase, 1.4 U/ml enolase, 0.25 mM β-NADH, 5 mM 3-phosphoglycerate; (10) for enolase: 100 mM Tris-HCl pH 7.4, 10 mM MgCl2, 2 mM ADP, 5U/ml pyruvate kinase, 55U/ml lactate dehydrogenase, β-NADH, 1 mM 2- phosphoglycerate; (11) for pyruvate kinase (PK): 50 mM Imidazol, 100 mM KCl, 2 mM MgCl2, 5 U/ml lactate dehydrogenase, 1 mM ADP, 0.25 mM β-NADH, 1 mM phosphoenolpyruvate. The activity of each enzyme was normalized by protein content and the result was expressed as nmol of product/ minute × µg of protein.

### Glutaminase activity

Glutaminase activity was performed using Ammonia Assay Kit (Sigma Cat. AA0100) following manufactured specifications. Statistic were done by nonparametric one-way ANOVA on GraphPad Prism 6.0. The assays is based on the quantification of ammonia, which reacts with α-ketoglutarate and NADPH in the presence of L-glutamate dehydrogenase, to form L-glutamate and NADP^+^. The reaction is followed by the decrease in the absorbance at 340 nm. The enzyme activity is expressed as a function of the total protein concentration in the whole cell lysate.

### Statistics

All graphs and statistics for western blot, enzymatic activities, RT-qPCR and oxygen consumption experiments were done on GraphPad Prism 6.0 and 95% of confidence level. The evaluation of statistic significant between two groups were done using unpaired t-test. In the graphs, we show as error the standard deviation (SD). Statistical significance was calculated using SD values.

Univariate analysis in Fig. 1 was done by multiple t-test and applying the Holm-Sidak method for multiple comparisons correction with alpha = 0.05. The multiple t-test was generated on GraphPad Prism version 7.00 for Windows (GraphPad Software, La Jolla California USA).

## Electronic supplementary material

Supplementary Information
