# Supplementary material for: Metabolomic Analysis Reveals Vitamin D-induced Decrease in Polyol Pathway and Subtle Modulation of Glycolysis in HEK293T Cells
Source: Sci Rep. 2017 Aug 25;7:9510. doi: 10.1038/s41598-017-10006-9 (PMC5573350; doi:10.1038/s41598-017-10006-9)
Supplement: Supplementary file 1 — Supplementary Information [file 41598_2017_10006_MOESM1_ESM.pdf]

Supplementary Material

Metabolomic Analysis Reveals Vitamin D-induced Decrease in Polyol Pathway and Subtle Modulation of Glycolysis in HEK293T Cells.

Santos GC[1,2†]; Zeidler JD[1]; Pérez-Valencia JA[1]; Sant'Anna-Silva ACB[1]; Da Poian, AT[1], El-Bacha T[2,3]; Almeida FCL[1,2†]

[1]Federal University of Rio de Janeiro; Institute of Medical Biochemistry Leopoldo de Meis

[2]Federal University of Rio de Janeiro, National Center for Structural Biology and Bioimaging (CENABIO) / National Center for Nuclear Magnetic Resonance (CNRMN).

[3]Federal University of Rio de Janeiro; Institute of Nutrition Josué de Castro.

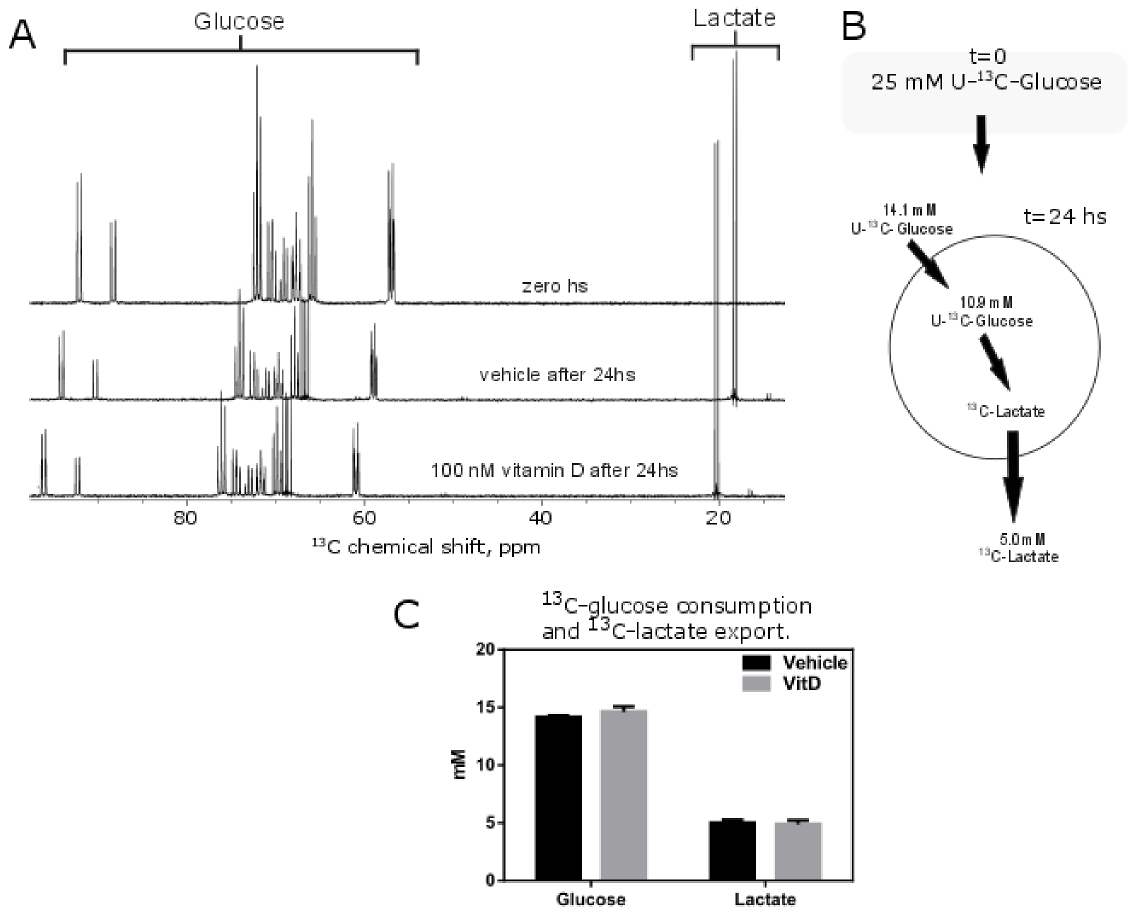

Supplementary Figure 1: ^{13}C-Glucose destination in HEK293T cells. HEK293T cells were treated by 100 nM of vitamin D (or vehicle as control) for 24hs. The cell culture medium was then replaced by DMEM containing 25 mM of ^{13}C-glucose (uniformly labelled, $^{13}C_6$) and 100 nM of vitamin D (or vehicle as control). It was incubated for another 24hs. After that, the growth medium was analyzed by NMR spectroscopy ^{13}C direct acquisition. The experiment was repeated 3 times for each condition. (A) The figure shows one representative ^{13}C-spectrum at time zero (t=0) (25 mM ^{13}C-glucose), and at time 24 hs (t=24hs) of vehicle and vitamin D-treated cells. We measured the integral (area) of all glucose peaks to evaluate, at t=0, the area (integral value) per ^{13}C. (B) Based on this value we quantitated the peak area of ^{13}C-glucose and ^{13}C-lactate at t=24 hs. At t=0 there were 25 mM of ^{13}C-glucose outside the cell and zero inside the cell. After 24hs (t=24hs) we measured 14.1 mM of ^{13}C-glucose and 5.0 mM of ^{13}C-lactate outside the cells. We calculated that 10.9 mM of ^{13}C-glucose entered the cell. ~23 % of the glucose (5.0 mM/10.9 mM) that entered the cells were converted and exported as ^{13}C-lactate. (C) The bar graph shows the measured values of ^{13}C-glucose and ^{13}C-lactate after 24 hs in the cells treated with vitamin D and vehicle. No significant difference was observed in ^{13}C-glucose consumption and ^{13}C-lactate accumulation with vitamin D treatment. Error bars represent S.E.M of biological triplicate, with 95% CI. Based on the presented data we calculated the cell specific rate for glucose uptake of 4.5 pmoles/cell/h. This data is compatible with the literature[1]. We also calculated the cell specific rate for lactate accumulation, which was 2.1 pmoles/cell/h.

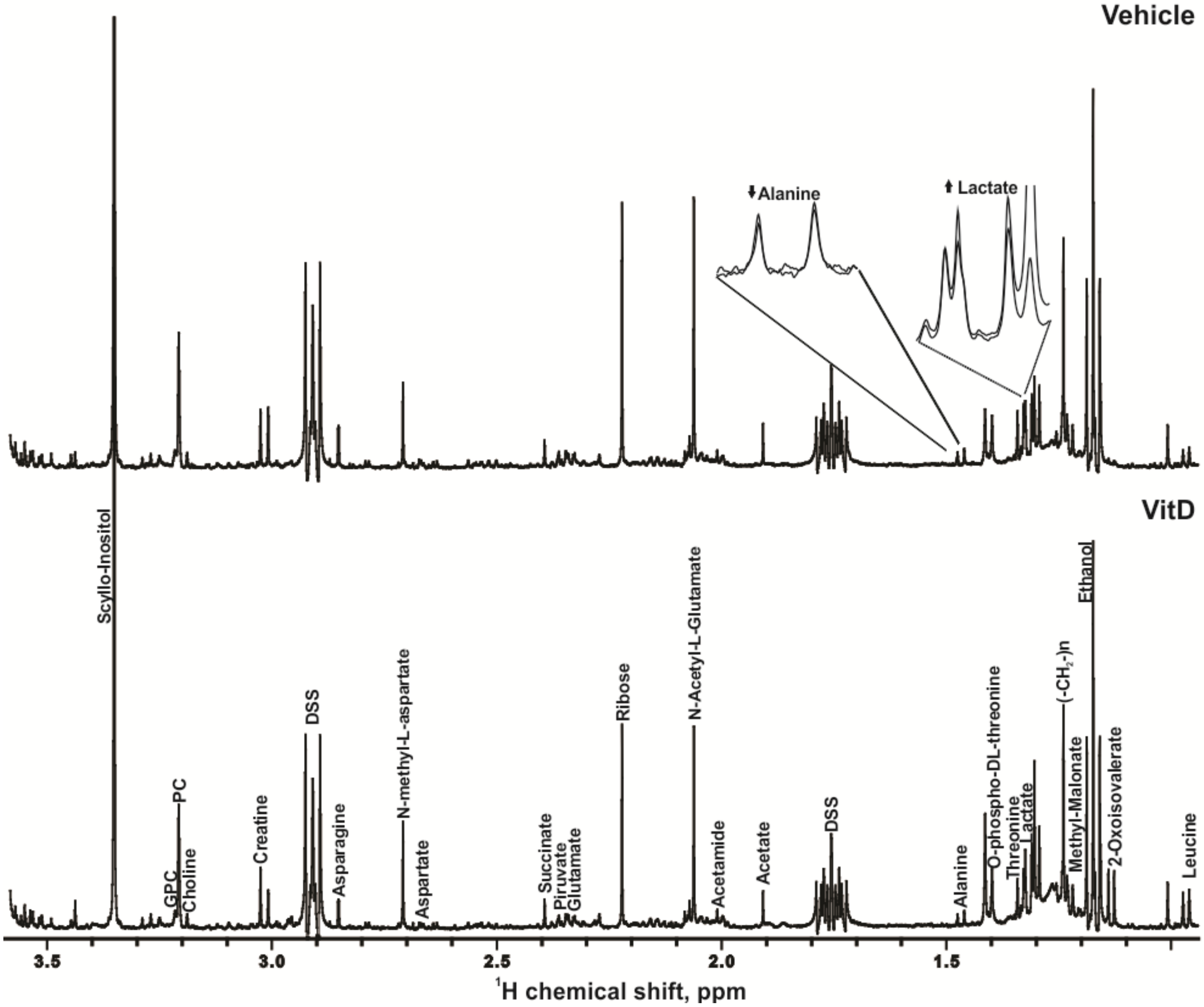

Supplementary Figure 2. 1D ^{1}H NMR spectra from HEK293T cell polar extract of aliphatic region. Lactate and alanine were showed up with vehicle and vitamin D treatment spectra aligned. Arrows ↑↓ indicate metabolites increased or decreased, respectively, by vitamin D treatment. PC- Phosphocholine; GPC- Glycerophosphocholine; DSS- 4,4-dimethyl-4-silapentane-1-sulfonic acid.

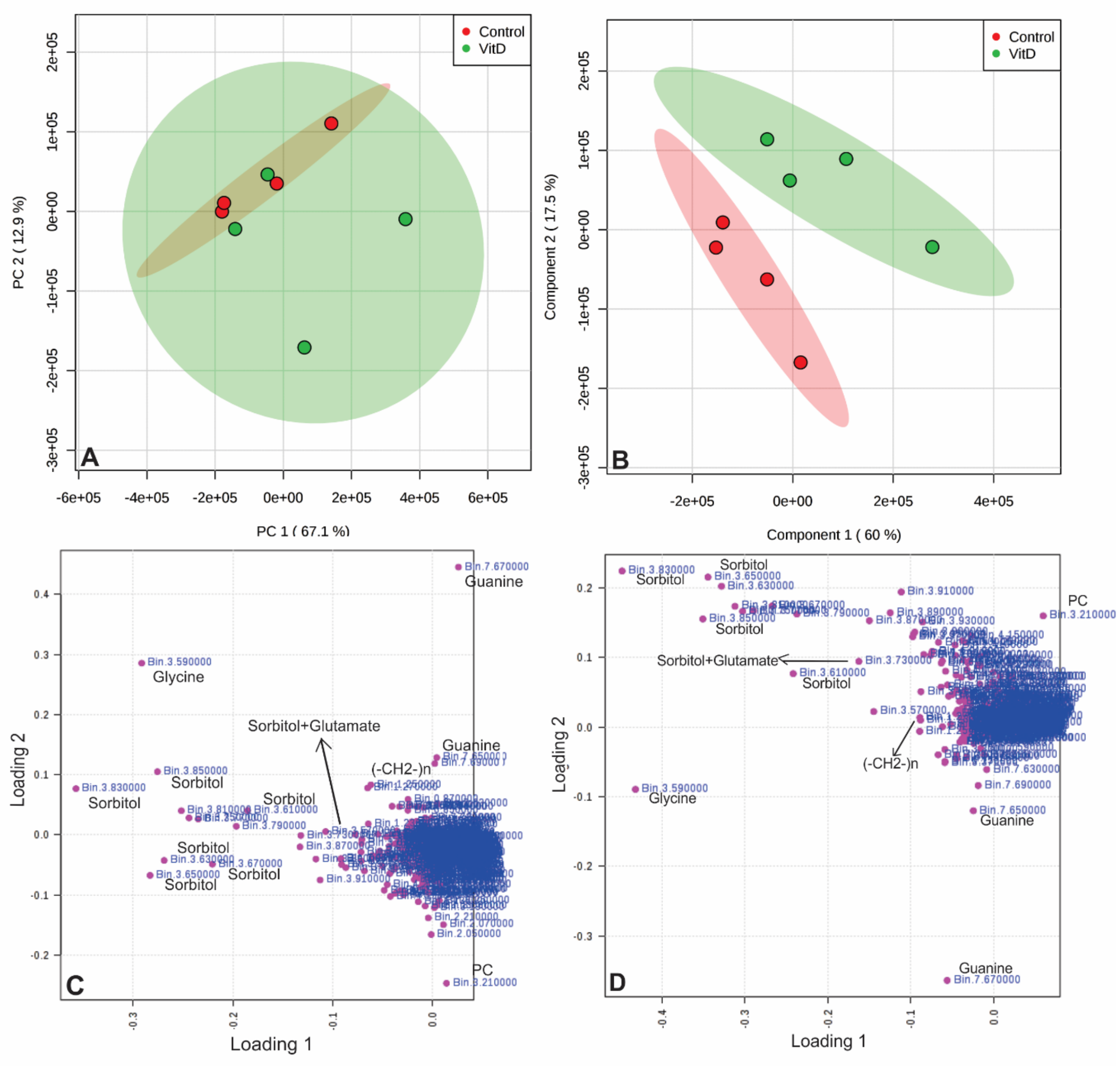

Supplementary Figure 3: **Comparison of the supervised (PLS-DA) and unsupervised (PCA) multivariate analysis of control and vitamin D-treated HEK293T cells.** (A) PCA score-plot shows a reasonable but not strong class discrimination. (B) PCA loading plot highlights important metabolites for class discrimination, as sorbitol, glycine, guanine and phosphocholine. (C) PLS-DA score plot shows strong class discrimination and (D) PLS-DA loading plot put in evidence the same metabolites, shown in PCA, evidencing that, although the class discrimination is not strong in PCA analysis, and could always be overfitted in the supervised PLS-DA, the important metabolites hierarchy is very similar in the supervised and unsupervised analysis: sorbitol, glycine, glutamate, guanine, (-CH$_2$-)n and phosphocholine (PC). Figure was created using the software MetaboAnalyst [2].

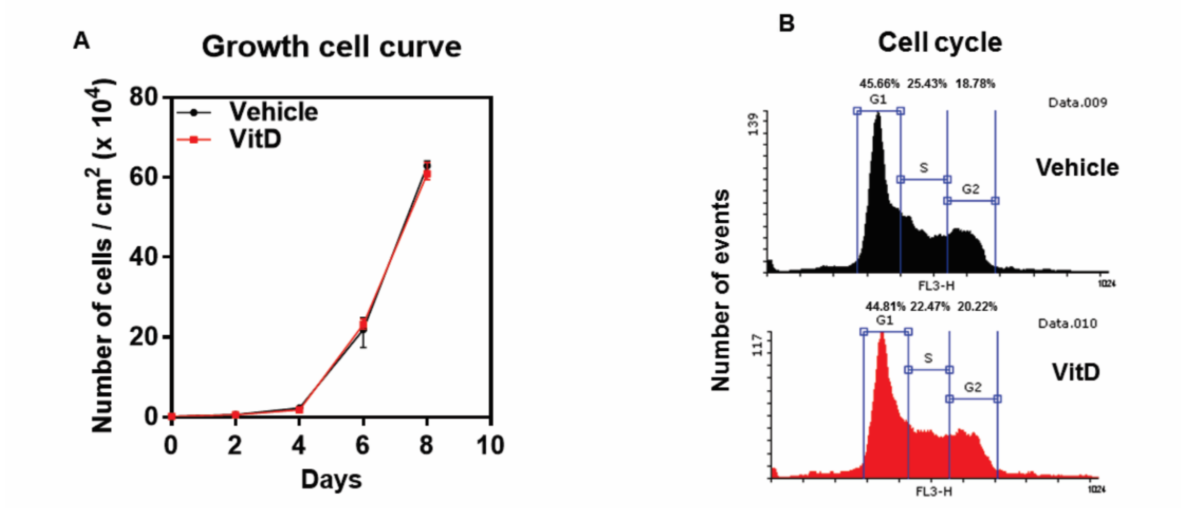

Supplementary Figure 4: Vitamin D do not change cell proliferation and cell cycle. (A) HEK293T cells were treated with 100 nM vitamin D or vehicle before fixed and stained with propidium iodide. The marks in the histograms of flow cytometry indicate cells with DNA content correspondent to G1, S and G2 cell cycle phases, as indicated. (B) Growth curves with HEK293T cells treated with 100 nM vitamin D or vehicle. The medium with the treatment was renewed each other day. The points are represented as mean ± SD n=3 and the curve is representative of three independent experiments.

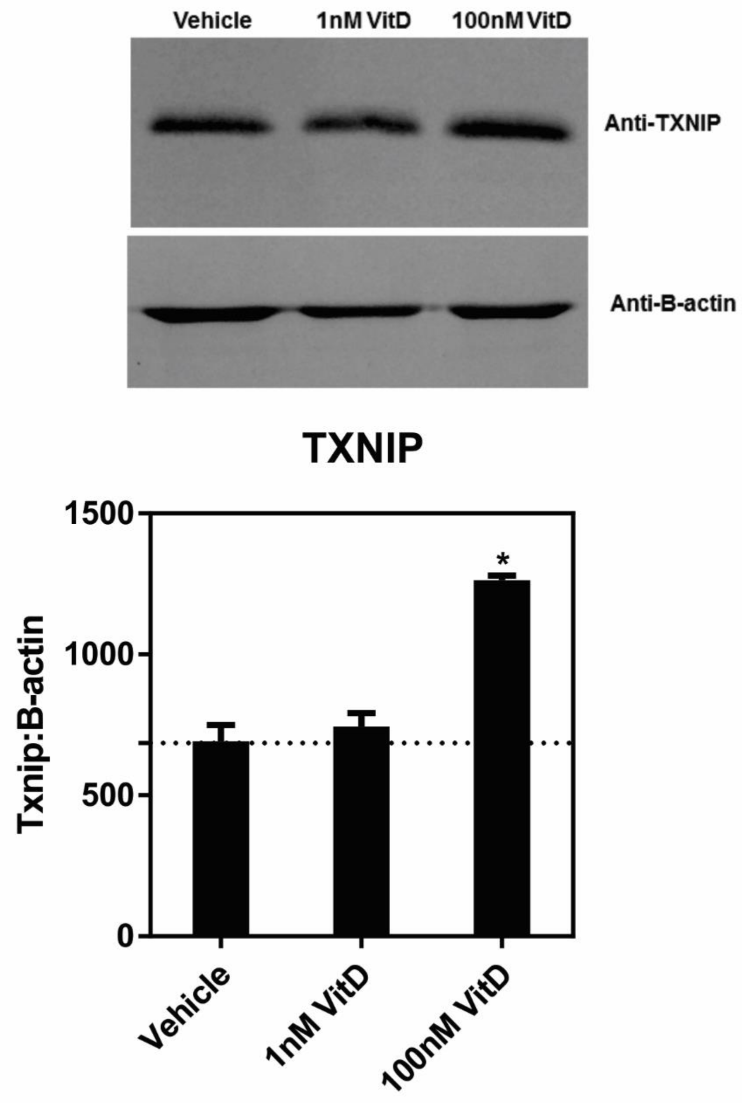

Supplementary Figure 5: Vitamin D leads TXNIP overexpression. Protein TXNIP was overexpressed by 24hs vitamin D treatment. Western-Blot of HEK293T whole-cell lysate, were repeated 2 times, with antibody anti-TXNIP and anti-β-actin. The graphic below represents densitometry (Image J®) of western-blot bands above. Error bars correspond to SD of biological duplicate. Statistical significance was evaluated using unpaired t-test (*$p<0.05$) according Material and Methods. β-actin is the internal control of constitutive expression. Densitometry of TXNIP and β-actin bands were independent, resulting from the same sample but different gels and antibodies. TXNIP:β-actin (y-axis) represent the normalized increase of TXNIP expression comparing vehicle and vitamin D, but should not be seen as the actual excess of TXNIP over β-actin.

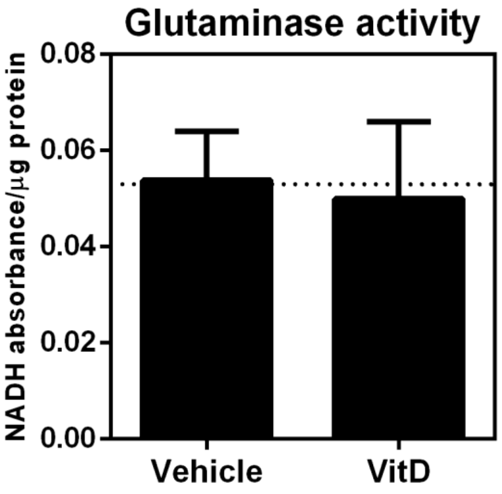

Supplementary Figure 6: Vitamin D do not change glutaminase activity. Bar graph shows the glutaminase activity measured in the whole cell lysate after treatment with 100 nM of vitamin D or vehicle. The error bars refer to the standard deviation (SD) of six independent experiments for each condition.

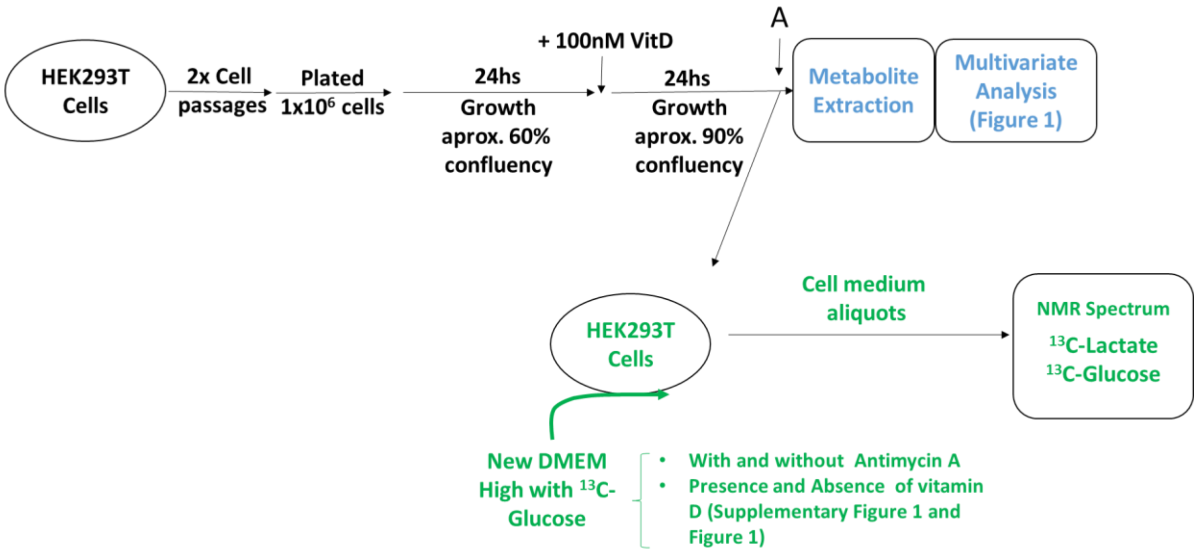

Supplementary Figure 7. Experimental design for the growth of HEK293T cells in high glucose medium. HEK293T cells were stored at -80 ºC. Cells were thawed from -80ºC and grown in DMEM with 25 mM of glucose for two passages. After the two passages, $1x10^6$ cells were plated in 100 mm petri dishes and grown for 24 hs. After the 24 hs the cells show approximately 60 % of confluency. At this point we added 100 nM of Vitamin D solubilized in 95% ethanol. For every experiment, we run a control experiment with the vehicle. After that we let the cells grow for another

24 hs reaching approximately 90% of confluency. The experimental design up to point A is common for all experiments.

For the multivariate analysis (Figure 1 and Supplementary Figure 2 and 3, blue), we pelleted the cells immediately for metabolite extraction.

For the experiments to measure the glucose fate (Supplementary Figure 1 and Figure 2, green), the cell medium was changed (after point A) by fresh DMEM containing 25 mM of ^{13}C-uniformly-labelled-glucose (#CLM-1396; Cambridge Isotope), with or without antimycin A (1 mg/mL) for 180 minutes.

Supplementary Table 1: Multivariate analysis data and VIP-scores for each bucket. Table shows the ^{1}H chemical shift values in ppm scale (δH), PLS-DA loading factors on components 1 (comp 1), 2 (comp 2) and 3 (comp 3), and VIP-scores on PLS-DA component 1. Table shows only buckets with VIP-scores higher than 0.2.

δH (ppm)	Comp 1	Comp 2	Comp 3	VIP score - Comp 1
3.59	-0.4322	-0.0896	0.00801	8.5248
3.83	-0.4484	0.2243	0.07134	4.6703
7.67	-0.0558	-0.3641	0.33934	3.9264
3.85	-0.3509	0.15543	0.12038	3.667
3.65	-0.3447	0.21555	-0.0844	3.2197
3.61	-0.2418	0.07676	-0.0156	3.1398
3.63	-0.3283	0.20248	-0.0552	3.1027
3.81	-0.3121	0.17363	0.0504	3.0138
3.75	-0.3028	0.16641	0.0284	2.9746
3.21	0.06015	0.15984	-0.1259	2.8918
3.77	-0.2897	0.16719	0.04221	2.686
3.67	-0.2671	0.17418	-0.0602	2.4134
3.57	-0.1445	0.02238	-0.0841	2.3611
3.37	-0.0586	-0.051	-0.1239	2.0188
3.33	-0.0586	-0.0322	-0.1983	1.9718
3.79	-0.2373	0.16241	0.05011	1.8452
7.29	-0.0666	-0.0397	-0.0238	1.7353
6.15	-0.0585	-0.0494	-0.0279	1.6977
3.73	-0.1625	0.09447	-0.0121	1.5862
1.25	-0.089	-0.0061	0.0518	1.5854
7.65	-0.0242	-0.1202	0.06703	1.5542
1.23	-0.088	0.01	-0.0298	1.5179
0.87	-0.0444	-0.0396	-0.0048	1.3999
1.27	-0.0887	0.01372	0.06145	1.3451
7.31	-0.0445	-0.0455	-0.003	1.3135
3.55	-0.0615	0.00056	-0.0971	1.2484
4.15	-0.027	0.13177	-0.0157	1.2104
0.89	-0.0323	-0.0422	-0.0144	1.2074
8.53	0.02388	0.07167	-0.0837	1.1603
3.19	0.02099	0.07196	-0.081	1.1364
0.85	-0.0399	-0.0209	-0.0121	1.1291
3.45	-0.0404	-0.0217	-0.067	1.1144
3.49	-0.0379	-0.0171	-0.0877	1.0871
1.41	-0.0462	0.11807	0.09023	1.0274
3.39	-0.0515	0.00408	-0.0667	1.0011
1.63	-0.0341	-0.0204	-0.0288	0.98896
1.61	-0.0335	-0.0173	-0.0342	0.97768

4.27	-0.0162	0.10215	-0.0382	0.97096
4.25	-0.0338	0.12188	-0.0102	0.96327
3.47	-0.0317	-0.0167	-0.0838	0.96298
1.75	-0.0352	-0.0138	-0.0322	0.91828
7.69	-0.0184	-0.084	0.1026	0.91702
8.25	0.02388	0.05475	-0.0939	0.90833
3.69	-0.0875	0.05091	-0.0319	0.90476
4.17	-0.0374	0.12423	-0.0228	0.90429
0.91	-0.0237	-0.0234	-0.0466	0.90346
1.59	-0.0351	-0.0088	-0.0307	0.89846
3.03	0.00816	0.07224	-0.0918	0.89482
0.83	-0.0319	-0.0114	-0.0251	0.89223
1.53	-0.0313	-0.0068	-0.0397	0.87654
1.65	-0.0323	-0.0088	-0.0359	0.84181
1.73	-0.0377	-0.0034	-0.0452	0.83754
6.13	-0.0268	0.07888	-0.0856	0.82246
1.55	-0.0349	-0.0002	-0.0324	0.82082
1.57	-0.036	0.00153	-0.0325	0.80104
7.63	-0.0081	-0.061	0.01185	0.79888
1.51	-0.0349	0.0006	-0.0301	0.79256
8.27	0.01955	0.04713	-0.0733	0.7886
3.41	-0.0368	0.00283	-0.0804	0.7821
1.79	-0.0292	-0.0082	-0.0342	0.76642
1.77	-0.0314	-0.0039	-0.0354	0.76049
1.91	-0.0381	0.01267	-0.1029	0.75637
3.31	-0.0155	-0.0136	-0.1163	0.73499
1.81	-0.0294	-0.0041	-0.0367	0.72962
7.61	-0.0113	-0.0402	-0.0119	0.71305
4.23	-0.016	0.08812	-0.0647	0.71155
1.67	-0.0312	0.00109	-0.0369	0.70651
4.37	0.00213	0.06258	-0.0545	0.70607
4.21	-0.0183	0.08976	-0.0675	0.6994
1.95	-0.0228	-0.0049	-0.0643	0.68937
7.59	-0.0152	-0.0302	-0.0121	0.68119
2.33	-0.0007	0.06528	-0.0584	0.68021
6.81	0.0161	0.04104	-0.0641	0.6671
4.29	-0.0308	0.09233	-0.0003	0.66498
1.49	-0.033	0.00869	-0.0359	0.65357
1.93	-0.0225	-0.0021	-0.0705	0.65121
0.99	-0.0247	-0.0062	-0.0397	0.64705
3.91	-0.1112	0.19411	0.03379	0.64421
3.51	-0.0307	0.0069	-0.0964	0.64266
1.69	-0.0318	0.00507	-0.0349	0.64195
4.39	-0.0011	0.06032	-0.0468	0.63213

1.97	-0.0272	0.00391	-0.0602	0.63097
1.83	-0.0302	0.00371	-0.0339	0.62923
3.43	-0.0345	-0.0006	-0.0418	0.62632
1.37	-0.0454	0.01948	-0.0079	0.62563
5.97	0.01594	0.04385	-0.0897	0.62214
2.35	0.00503	0.05483	-0.0644	0.6184
0.81	-0.0229	-0.0015	-0.0328	0.6111
4.13	-0.0365	0.09681	-0.0086	0.61007
1.39	-0.0457	0.10301	0.02136	0.60452
4.19	-0.0284	0.09533	-0.0651	0.58789
1.71	-0.0345	0.01493	-0.0411	0.58307
3.93	-0.0855	0.15101	0.03572	0.54405
0.93	-0.0209	0.00276	-0.0545	0.5385
7.45	-0.025	-0.0116	0.00598	0.53429
4.31	-0.032	0.08224	0.01479	0.53416
3.87	-0.1498	0.15294	0.04124	0.53301
7.57	-0.0154	-0.0191	-0.0079	0.52978
1.47	-0.0342	0.02152	-0.053	0.52894
3.53	-0.0283	0.01376	-0.107	0.51808
1.35	-0.0432	0.02418	-0.006	0.51348
2.15	0.005	0.05071	-0.085	0.5084
1.13	0.02403	-0.0159	0.02754	0.50652
1.15	0.01993	0.01575	-0.0616	0.5023
1.85	-0.0359	0.01856	-0.0165	0.49054
6.83	0.01148	0.03346	-0.0595	0.48948
1.45	-0.0357	0.02615	-0.0467	0.48384
7.25	-0.0198	-0.0024	-0.0322	0.4802
7.95	0.00723	0.03844	-0.0575	0.46154
2.25	-0.017	0.00582	-0.0648	0.4587
2.61	-0.012	-0.0032	-0.0582	0.45177
4.05	-0.0663	0.12178	0.00563	0.44992
2.73	-0.0184	0.00283	-0.0549	0.447
3.25	-0.0293	0.01566	-0.0777	0.44629
4.35	-0.0075	0.05676	-0.0582	0.43966
1.89	-0.0375	0.02699	-0.0436	0.43606
2.87	-0.0087	-0.0007	-0.0757	0.42782
1.87	-0.0329	0.02177	-0.0316	0.42693
4.33	-0.0164	0.06169	-0.0291	0.42135
2.59	-0.0159	0.00583	-0.0588	0.41655
6.03	-0.009	-0.0107	-0.0369	0.41527
2.43	-0.013	-0.0023	-0.0336	0.41489
3.07	-0.0213	0.01028	-0.0635	0.41473
2.75	-0.0142	0.00096	-0.0552	0.41296
6.17	-0.0318	0.01377	-0.0102	0.41043

3.01	0.00674	0.04561	-0.1034	0.40428
2.45	-0.0115	-0.0015	-0.0549	0.40388
2.19	-0.013	0.01078	-0.0998	0.40171
2.23	0.00864	-0.0168	-0.1515	0.39509
8.43	-0.0106	-0.0053	-0.0475	0.39193
7.97	0.00929	0.03245	-0.0679	0.3915
2.47	-0.0032	-0.0092	-0.0713	0.38471
1.03	-0.0094	-0.0021	-0.064	0.38369
7.19	-0.014	-0.004	-0.0225	0.37981
5.85	-0.0105	-0.002	-0.0467	0.37085
2.71	-0.0495	0.04696	-0.02	0.36887
7.33	-0.0373	0.02193	-0.0022	0.36656
6.89	-0.0136	-0.0017	-0.0289	0.36619
8.45	-0.0065	-0.0071	-0.0517	0.36465
1.21	-0.0274	0.07232	-0.0708	0.36281
3.29	-0.0096	0.00104	-0.1012	0.36088
6.91	-0.0102	-0.0042	-0.0291	0.36045
6.07	-0.0539	0.04446	0.00488	0.35813
1.31	-0.0739	0.10777	0.08031	0.35539
5.83	-0.0118	0.00183	-0.0481	0.34621
1.99	-0.0278	0.02436	-0.0573	0.34589
2.41	-0.0115	0.00491	-0.055	0.34586
7.15	-0.0015	-0.0124	-0.0344	0.33253
7.17	-0.01	-0.0032	-0.0326	0.33134
8.07	-0.0086	-0.0007	-0.0409	0.32812
2.77	-0.0111	0.00313	-0.0561	0.32366
2.03	-0.0181	0.02019	-0.1075	0.32282
6.09	-0.0292	0.01984	-0.0273	0.31823
8.41	-0.0052	-0.0051	-0.0454	0.31332
8.13	0.00525	0.02798	-0.0478	0.31245
5.99	0.01042	0.02383	-0.0677	0.312
6.93	-0.0086	0.00113	-0.0427	0.31143
2.13	0.00148	0.04173	-0.09	0.3108
1.33	-0.0555	0.0605	-0.0341	0.31062
6.05	-0.0145	0.00518	-0.0353	0.30657
1.07	-0.0083	0.00371	-0.0612	0.30442
2.95	-0.0173	0.04319	0.00834	0.30275
8.03	-0.0064	-0.0026	-0.0389	0.30157
7.71	-0.0172	-0.0158	0.05077	0.29989
7.35	-0.0632	0.05743	0.02376	0.29948
2.27	-0.0267	0.02447	-0.0463	0.29296
5.89	-0.0125	0.00393	-0.0347	0.29165
2.53	0.00533	0.02903	-0.0754	0.2904
7.81	-0.0103	0.00079	-0.0185	0.28962

2.81	-0.0057	-0.0001	-0.0579	0.28843
7.27	-0.0258	0.01666	-0.0193	0.28771
8.17	-0.0005	-0.0101	-0.0509	0.28312
5.87	-0.014	0.00788	-0.0366	0.28142
7.83	-0.0046	-0.0044	-0.0278	0.2792
1.43	-0.041	0.03718	0.00599	0.27759
1.19	0.00335	0.02095	-0.0269	0.27745
5.95	0.00944	0.0267	-0.0854	0.27717
5.81	-0.0089	0.00314	-0.0463	0.27698
3.23	-0.0212	0.01319	-0.0593	0.27321
4.11	-0.0386	0.07171	0.00111	0.26875
8.05	-0.01	0.00496	-0.0319	0.26866
2.97	-0.0098	0.03583	-0.0184	0.26743
2.63	-0.0095	0.01106	-0.0786	0.26545
5.67	-0.0116	0.00564	-0.0389	0.2579
7.93	0.00787	0.02573	-0.0618	0.25288
5.65	-0.0055	0.0034	-0.0558	0.25244
3.11	-0.0186	0.01621	-0.0524	0.24845
2.89	-0.0067	0.01114	-0.0713	0.24266
2.09	0.00278	0.03629	-0.1134	0.23737
2.29	-0.0256	0.02508	-0.0386	0.23259
5.75	-0.0076	0.0084	-0.0555	0.22433
7.75	-0.0088	-0.0054	0.01022	0.22154
6.25	-0.0209	0.01619	-0.0121	0.22124
5.73	-0.006	0.00266	-0.0521	0.22085
5.69	-0.0066	0.00166	-0.0455	0.21953
3.09	-0.0203	0.01905	-0.0493	0.21844
2.69	-0.0153	0.0235	-0.0788	0.21842
2.93	-0.0095	0.02557	0.02332	0.21831
2.51	0.00766	0.0233	-0.0915	0.21676
5.79	-0.0094	0.00608	-0.0338	0.21502
7.73	-0.0121	-0.0057	0.02861	0.21142
7.87	-0.0125	0.00555	-0.0143	0.21008
2.39	-0.0127	0.01934	-0.0575	0.20902
1.05	-0.0019	0.00307	-0.0707	0.20813
8.29	0.00642	0.01889	-0.0516	0.20413
7.43	-0.0251	0.01671	0.0066	0.20209
5.71	-0.0096	0.00496	-0.0362	0.20200

Table S2. Metabolites with the most discriminating power between Vitamin D treated and control HEK293T cells, according to PLS-DA multivariate and univariate statistics[a]

Metabolic pathway	Metabolite	Status (LV1)*	δH (ppm)	LV1	LV2	LV3	VIP score - Comp 1	Univariate Analysis
Aminoacid	Glycine	↓	3.59	-0.43218	-0.08956	0.00801	8.52480	↓ -44 %*
	Creatine	↑	3.03	0.00816	0.07224	-0.09178	0.89482	↑ +21 %
	Alanine	↓	1.47	-0.03418	0.02152	-0.05304	0.52894	↓ -10 %
Polyol	Sorbitol	↓	3.83	-0.44838	0.22430	0.07134	4.67030	↓ -17 %*
PPP[1]	Guanine	↓	7.67	-0.05578	-0.36407	0.33934	3.92640	↓- 49 %*
	Orotate	↓	6.15	-0.05848	-0.04938	-0.02786	1.69770	↓ -28 %
Glutamate	Sorbitol+Glutamate	↓	3.75	-0.30278	0.16641	0.02840	2.97460	↓ -16 %*
Fatty acid	(-CH2-)n	↓	1.25	-0.08897	-0.00608	0.05180	1.58540	↓ -15 %
Choline	Phophocholine	↑	3.21	0.06015	0.15984	-0.12592	2.89180	↑ +56%
	Choline	↑	3.19	0.02099	0.07196	-0.08100	1.13640	↑ +34%
TCA[2] cycle	Succinate	↓	2.39	-0.012662	0.019336	-0.057528	0.20902	●-4 %
Others	Acetate	↓	1.91	-0.038141	0.012673	-0.10294	0.75637	↓ -19 %
	Formate	↓	8.43	-0.01061	-0.00527	-0.04747	0.39193	↓ -22 %
	Piruvate	↑	2.35	0.005034	0.054827	-0.064387	0.61840	↑+12 %
	Lactate	↓	1.31	-0.073854	0.10777	0.080312	0.35539	● + 4 %
***Status after vitamin D treatment; 1-Pentose Phosphate Pathway; 2-Tricarboxylic Acid;↑Increased; ↓Decreased; ● Constant**								

[a]PLS-DA model was validated by the permutation test, B/W-ratio and cross-validation by leave-one-out method. Metabolites were ranked according to their VIP-scores on component 1 and the PLS-DA regression coefficients (loading vectors-LV) on components 1, 2 and 3 (LV1, LV2 and LV3), as described in Material and Methods. ↑ indicates an increase and ↓ indicates a decrease in metabolites concentration in vitamin D treated cells compared to control cells according to the PLS-DA LV1. The column of univariate analysis refers to the percentage of change of each metabolite bucket area upon treatment with vitamin D. Up and down arrows indicate increase oo decrease of the metabolite content. Filled circle indicate that the metabolite concentration was approximately constant.
[1]PPP: Pentose Phosphate Pathway; [2]TCA: Tricarboxylic Acid

References:

1. Pörtner, R., Bohmann, A., Lüdemann, I. & Märkl, H. Estimation of specific glucose uptake rates in cultures of hybridoma cells. *J. Biotechnol.* **34,** 237–246 (1994).

2. Xia, J., Sinelnikov, I. V, Han, B. & Wishart, D. S. MetaboAnalyst 3.0--making metabolomics more meaningful. *Nucleic Acids Res.* **43,** W251-7 (2015).
